# Histidine alleviates Hashimoto’s thyroiditis via the neutrophil extracellular traps-NF-κB signaling pathway

**DOI:** 10.1038/s41598-026-45671-2

**Published:** 2026-03-26

**Authors:** Tingting Ding, Yuling Wang, Lijian Zhang, Xinrui Zhou, Tingwei Cheng, Qiong Wang, Lei Yu, LanGen Zhuang, GuoXi Jin

**Affiliations:** 1 Department of Endocrinology, The First Affiliated Hospital of Bengbu Medical University, Bengbu, Anhui P.R. China; 2 Anhui Province Key Laboratory of Basic and Translational Research of Inflammation-related Diseases, Bengbu, Anhui P.R. China

**Keywords:** Hashimoto’s thyroiditis, Metabolomics, Histidine, Neutrophil extracellular traps (NETs), NF-κB signaling pathway, Biochemistry, Diseases, Endocrinology, Immunology, Medical research

## Abstract

Metabolite abnormalities are potentially implicated in pathogenesis of Hashimoto’s thyroiditis (HT). An in-depth study of the relationship between HT and small molecule metabolites, as well as its pathogenesis, can help enhance the diagnosis, prognosis, and treatment of HT patients. We used metabolomics to analyze changes in serum metabolite levels in 20 HT patients and 20 healthy controls (HC) and the most significant differentially expressed metabolite was analyzed. Western blot and qPCR were used to measure the expression of histidine decarboxylase (HDC) and histamine receptor 1 (H1R). Increasing concentrations of histidine were used to treat neutrophils and observe the effect on neutrophil extracellular traps (NETs) synthesis. Histidine treated neutrophils were co-culture with thyroid follicular cells to study the protective effects of histidine on HT thyroid follicular cells and related mechanisms. A total of 48 differentially expressed metabolites were found. Histidine exhibited reduced expression levels in HT patients, displaying the most significant discrepancy (*p* < 0.001). ROS and NETs were increased and HDC, H1R, and histamine were upregulated in neutrophils upon stimulated. These effects were corrected by addition of histidine, in a dose dependent manner. In addition, in co-culture experiments, histidine enhanced expression of SOD and suppressed production of inflammatory cytokines IL-6 and TNF-α and NF-κB pathway in thyroid follicular cells, thereby inhibiting inflammation and oxidative stress. Histidine inhibits NETs synthesis and NF-κB signaling. Thus, histidine play an anti-inflammatory and antioxidant role by decreasing thyroid follicular cell inflammation in HT.

**Supplementary Information:**

The online version contains supplementary material available at 10.1038/s41598-026-45671-2.

## Introduction

Hashimoto’s thyroiditis (HT) is a common autoimmune disease that is characterized by elevated serum anti-thyroid peroxidase (TPO), and anti-thyroglobulin (TG) antibodies and thyroid lymphocyte infiltration^1^. In severe cases, patients can develop hypothyroidism. In addition, HT tends to occur in women and is a common cause of female infertility and abortion^2,3^. Furthermore, a study reported that HT may be a risk factor for thyroid cancer^4^. At present, the etiology and pathogenesis of HT are still not completely elucidated and warrants further studies.

Metabolomics is the best tool for studying metabolites and disease. Metabolites analyzed using metabolomics usually refer to small molecule metabolites with molecular weight < 1500 da, including but not limited to amino acids, lipids, organic acids, and some exogenous chemicals^5^. Some studies showed that metabolic abnormalities may be associated with HT. For example, Xu et al. found changes in serum metabolites in HT patients^4^. Fatty acids and lysine degradation pathways have been shown to have to be dysregulated in HT patients with^4^ normal thyroid function as well as in those with subclinical hypothyroidism^6^. In addition, it has been shown that there are changes in the amino acid metabolism profile between papillary thyroid carcinoma patients with and without HT^7^, showing that there is some correlation between HT and metabolism abnormalities in thyroid cancer. In summary, metabolic abnormalities participate to HT pathogenesis and active research on the relationship between metabolites and HT can help improve HT diagnosis, prognosis, drug screening, and personalized treatment.

A study showed that neutrophil extracellular traps (NETs) may participate in HT pathogenesis^8^. NETs are specialized network-like structures that are generated by neutrophils in response to stimulation, involving the production of reactive oxygen species (ROS), citrullinated histone 3 (CITH3), and myeloperoxidase (MPO). CITH3 and MPO serve as specific markers for NETs^9^. NETs formation is accompanied by neutrophil death and this new form of cell death differs from apoptosis and necrosis and is known as NETosis^10^. During NETosis, the release of nuclear DNA from neutrophils needs several key steps: (i) chromatin dencondensation via PAD4-mediated histone citrullination^11^; (ii) the nuclear envelope rupture through PKCa-mediated lamin B phosphorylation^12^ and CDK4/6-mediated lamin A/C phosphorylation^13^; (iii) cytoskeleton-related plasma membrane breakdown^14^.

ROS synthesis and PAD4 deamination are prerequisites for NET formation. Microorganisms and its products, cytokines, immune complexes, autoantibodies, and chemicals can induce neutrophils to release NETs and participate in the pathogenesis of many diseases^15^, including autoimmune diseases, such as rheumatoid arthritis, SLE, type I diabetes mellitus, and antiphospholipid syndrome (APS)^16–19^. Plasma NET products are significantly higher in HT patients than healthy controls and positively correlate with TG and TPO autoantibodies titers^8^. Thus, NETs play an important role in the pathogenesis of HT.

Interestingly, a recently study demonstrated that neutrophils deficient in histidine decarboxylase (HDC) can mediate NET synthesis through histamine receptor 1 (HR1) and histamine, thereby inducing cardiomyocyte death and increasing fibroblast activation^20^. This shows that there is an association between NETosis and histamine. At present, there are few studies on the correlation between NETs and HT metabolites. Therefore, herein we employed metabolomics to analyze the relationship between abnormal metabolites and NETs and attempted to study the possible pathogenesis of HT in an in-depth manner.

First, we conduced metabolomics of serum samples from HT patients and healthy controls (HC) and found that the most differentially expressed metabolite was histidine. Serum histidine was decreased in HT patients. Next, to examine the role of histidine in HT patients, we constructed a NETs model to study the effects of histidine on HT.

## Materials and methods

### Patients

20HT patients (HT) who visited the Endocrinology Outpatient Department of the First Affiliated Hospital of Bengbu Medical College from February 2023 to May 2023 were selected. In addition, 20 healthy controls (HC) were selected. Peripheral blood (around 5 mL) was collected from all subjects and the serum was prepared and stored at − 80 °C for analysis. All participants gave written informed consent to participate. This study was approved by the Clinical Medical Research Ethics Clerk Committee of the First Affiliated Hospital of Bengbu Medical College(approval no.: 2023YJS108). Table 1 shows the general clinical data of HT patients and HC. The inclusion criteria for HT patients were based on the “Chinese Guidelines for the Diagnosis and Treatment of Thyroid Diseases” in 2008.The selection criteria for HT were as follows: (a)palpationand thyroid color ultrasonography sh owed diffuse goiter with tough texture, especially in patients with enlargement of the vertebral lobe of the thyroid isthmus; (b)patients were positive for thyroid peroxidase antibody (TPOAb) and/or thyroglobulin antibody (TgAb); (c) thyroid fine needle aspiration cytology showed diffuse lymphocytic and plasma cell infiltration, and fibrosis of thyroid gland; (d)thyroid function was normal or TSH increased with or without decreased FT3 and/or FT4. Among these criteria, (a) (b) (d)were necessary conditions. This HT group consists of newly diagnosed patients who have not received drug treatment.

**Table 1:**
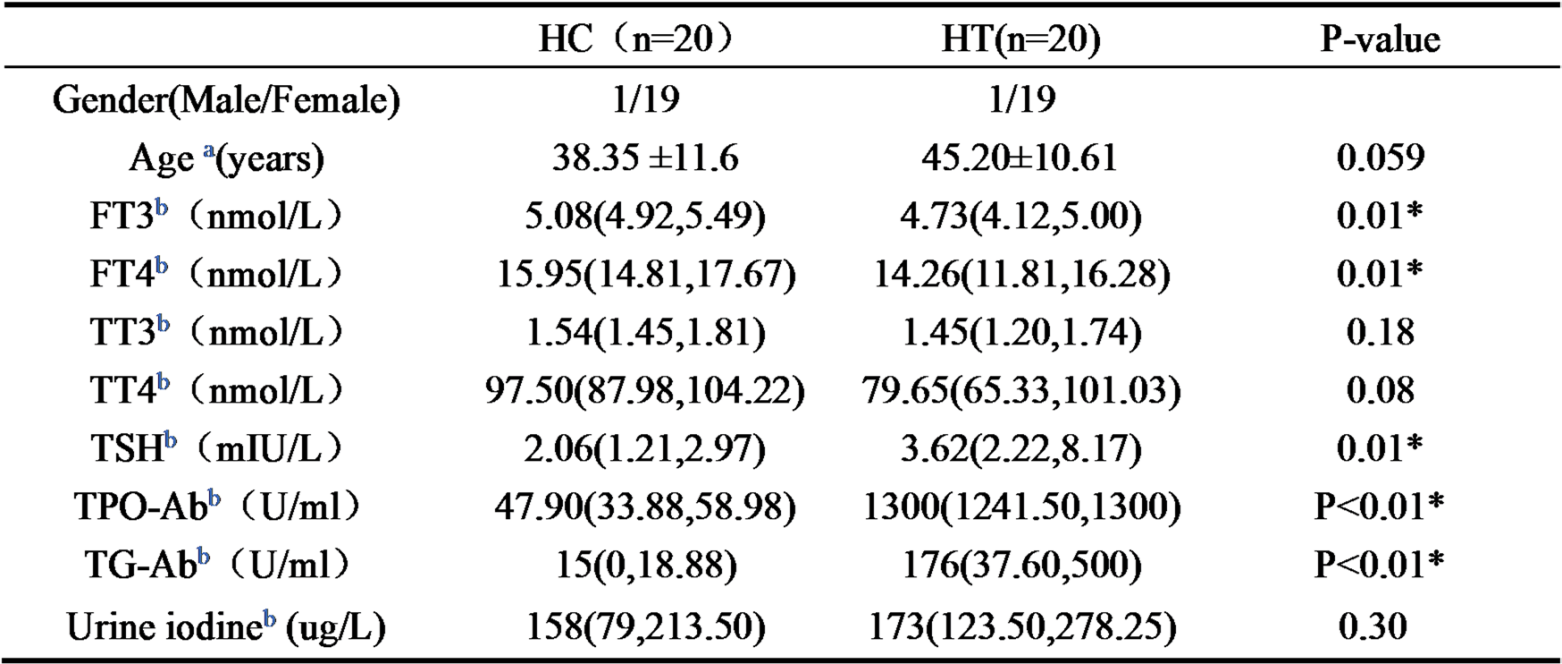
Clinical data of HC group and HT group.

All 20 patients with Hashimoto’s thyroiditis were newly diagnosed and had not taken any medication. A small number of patients with Hashimoto’s thyroiditis were diagnosed with hypothyroidism.

There was a significant difference in FT3,FT4,TSH, TPO-Ab, TG-Ab between the two groups (*p* < 0.05).

*represent *P* < 0.05. Normal Ranges: FT3:2.77–6.77.5pmol/L; FT4:10.43–24.43.32pmol/L; TT3:1.01–2.01.95nmol/L; TT4:55.34–160.34.88nmol/L; TSH:0.4–4.4.34mIU/L; TPO-Ab:<60U/ml; TG-Ab:<60U/ml.Pvalue: (usingtheMann‒WhitneyUtest).

^a^Mean ± SD.

^b^M(Q1,Q3).

The control group consists of people who were found to be healthy on physical examination. Inclusion criteria: (1) Normal thyroid function and absence of anti-thyroid autoantibodies; (2) Absence of thyroid disorder or autoimmune disease; (3) Absence of infectious diseases and normal hepatic and renal function, blood glucose, and blood lipids. Exclusion criteria: (1) Presence of other thyroid disorders and taking drugs that affect thyroid function; (2) Presence of other autoimmune diseases or tumors; (3) Taking immunosuppressants or glucocorticoids; (4) Infection, inflammation, or trauma due to various causes. All donors provided written informed consent for sample collection and subsequent analysis. Moreover, all experiments were performed in accordance with the relevant guidelines and regulations.

### Sample preprocessing

Serum samples were thawed on ice to minimize sample degradation. During the experiment, 20 µL of serum was transferred into a pre-cooled 96-well plate. The 96-well plate was placed in an Eppendorf epMotion workstation (Eppendorf Inc., Humburg, Germany) and 120 µL of methanol solution containing internal standards was automatically added to every well. The plate was vortexed for 20 min to mix evenly and then centrifuged at 4000 *g*/min for 20 min. The plate was taken out and placed on the workstation and 30 µL of supernatant was transferred into a new 96-well plate. 20 µL of freshly prepared derivatization reagent was added to every well and the plate was incubated at 30 °C for 60 min for derivatization. Next, 330 µL of ice cold 50% methanol solution was added to dilute the samples. The plate was centrifuged at 4 °C (4000 *g*, 30 min) and 135 µL of supernatant was transferred into a new 96-well plate. The plate was sealed for liquid chromatography mass spectrometry (LC-MS) analysis.

### Metabolomics

The Q300 reagent kit (metabolo-profile, Shanghai, China)^21^ was used for metabolomics. In this study, ultra performance liquid chromatography tandem mass spectrometry (UPLC-MS/MS, ACQUITYUPLC-XevoTQ-S, WatersCorp., Milford, MA, USA) was used to quantitate all target metabolites. The QuanMET software (v2.0, Metabo-Profile, Shanghai, China) was used to process the raw data files generated from UPLC-MS/MS. Peak acquisition, correction, and quantitation were carried out for each metabolite. Multivariate statistical analysis (Principal Component Analysis PCA, Partial least squares-Discriminant Analysis PLS-DA, Orthogonal Partial least squares-Discriminant Analysis OPLS-DA, VIP ≥ 1 and *P* < 0.05) and univariate statistical analysis (*P* < 0.05, |log2FC| >0) were used to screen for differentially expressed metabolites.

### Cell lines, reagents, antibodies

The human promyelocytic leukemia cell line (HL60) was purchased from Procell Life Science&Technology Co., Ltd., The human normal thyroid follicular cell line (Nthy-ori3-1) was purchased from the Chinese Academy of Sciences Cell Bank. Fetal bovine serum (A3160802, Gibco), 0.25% trypsin (B2048, BIOMEOICAL), 1640 (C11875500BT, Gibco), PS (15140122, Gibco), PBS (15140122, Gibco), methanol (10014118, Lushi), and DMF (Sigma) were purchased. CD11b antibody (379902, BioLegend) and CD66b antibody (305120, BioLegend) were purchased.

### Cell culture

Neutrophils were collected. 70 mM DMF was used to induce differentiation of HL60 into neutrophils. High frequency of CD11b+/CD66b+ indicates successful induction.

Our group has found that 20 mmol/L potassium iodide (KI) is the optimal concentration for the NETs model. (ref). Therefore, we used 20 mM KI to stimulate neutrophils for 2 h in this study. At the same time, 100 nM PMA was used to stimulate neutrophils for 2 h as positive control. Non-stimulated neutrophils were used as the negative control. The cells were divided into 3 groups, namely the control group, KI group, and PMA group.

The NETs model was co-cultured with normal thyroid follicular cells (Nthy-ori3-1). Different concentrations of histidine (0, 1, 5, and 10 mmol/L-His) were used to treat neutrophils for 24 h. Neutrophils were diluted to 2.5 × 10^5^ cells/mL and 2 mL of cell suspension was added to the lower chamber of a 6-well plate. Chambers were placed onto 6 well plates and 2.5 × 10^5^ treated Nthy-ori 3 − 1 cells were added to the upper chamber with a culture volume of 500 µL. The cell culture plate was incubated in an incubator for 24 h. The plates were used for subsequent functional studies.

### Immunofluorescence

We have supplemented the assessment of NETs formation by detecting the fluorescence co-localization of citH3 and MPO. Numerous studies using this methodology detect the formation of NETs. Many studies have used the same method to reliably detect the formation of NETs. Yi^22^ and Xu^23^ et al. have used MPO, CitH3, and DAPI staining to detect the formation of neutrophil extracellular traps in studies of liver cancer and lung cancer. Additionally, Xu et al.^24^ also used the same method to detect the levels of NETs in septic cardiomyopathy. To identify NETs, neutrophils were fixed using 4% paraformaldehyde and the mixture of primary antibodies MPO(ProteinTech Group, item number 66177-1-Ig, anti-mouse) and CITH3(ABclonal Biotech, item number A18298, anti-rabbit) was added. The mixture was incubated at 4 °C, overnight. After PBS washing, secondary antibodies were added. Alexa Fluor 488-labeled goat anti-rabbit IgG, GB25303, Servicebio (CitH3 secondary antibody) and CY3-labeled goat anti-mouse IgG, GB21301, Servicebio (MPO secondary antibody) were incubated at room temperature for 50 min. DAPI (Servicebio, G1012) was used for cell nucleus counterstaining and mounting was carried out using a fluorescence quencher (G1401 Servicebio). An upright fluorescence microscope (NIKON ECLIPSE C1) was used to acquire images (DAPI channel cell nucleus appeared blue, positive 488 channel appeared green, and positive CY3 channel appeared red). No negative or positive controls were conducted in this experiment.

### Western blot

RIPA lysis buffer (containing PMSF) was used to extract total protein and the BCA quantitation kit was used for total protein quantitation. SDS-PAGE electrophoresis was carried out before membrane transfer. Membranes were placed in TBST (containing 5% skimmed milk or BSA) and blocking was carried out at room temperature for 2 h. Next, the membranes were washed with TBST at 10 min×3 times. Primary antibody hybridization and secondary antibody hybridization were carried out. The primary antibodies used for hybridization were p-p65 (ABclonal AP0123 1:1000), p65 (ABclonal A19653 1:1000), p-IκBα (Thermo Fisher MA5-15087 1:1000), IκBα (ABclonal A1187 1:1000), GAPDH (Proteintech 60004-1-Ig 1:10000). The secondary antibody used for hybridization was goat anti-rabbit IgG-HRP (Absin catalog no.: abs20040 1:10000). After incubation with secondary antibody, the membranes were washed multiple times, and the development solution was added for fluorescence imaging. The protein quantitative analysis method involves using Fusion software to analyze the optical density values of the bands, with each band repeated three times. The relative expression level of the target protein ={ the target protein(Optical density value)/Internal Reference༈Optical density value༉} Western blot is three biological repeat experiments.

### Quantitative real-PCR

Total RNA from cultured cells was extracted using TRIzol and ultra-micro nucleic acid-protein analyzer was used to measure concentration. The SYBR Green Pro Taq HS premix qPCR assay kit from Accurate Biotechnology Co., Ltd was used for reverse transcription of total RNA. Real-time fluorescence quantitative PCR was used for cDNA amplification to analyze RNA expression, which was normalized to GAPDH expression. Table 2 shows the primer sequences. qPCR is three biological repeat experiments.

**Table 2:** Real-time PCR primers and conditions.

Gene	Genbank accession	Primer sequences (5’to3’)	Size(bp)	Annealing (℃)
Human GAPDH	2597	GGAGCGAGATCCCTCCAAAAT	197	61.6
		GGCTGTTGTCATACTTCTCATGG		
HDC	3067	CTCTGGTCAGAAACGACCCT	189	56.08
		GGGATGTCACAGTGAAACGG		
H1R	3269	GTCACAGTAGGGCTCAACCT	91	56.05
		AGAGGCTGACGATGTACAGG		

### Flow cytometry for measurement of neutrophil percentage

After 5 days stimulation with DMF, HL60 cells were collected by centrifugation and unstimulated HL60 cells were used as control to evaluate the effects of DMF on HL60 cells. A pre-cooled staining buffer was used to gently resuspend the cell pellet to a final concentration of 1 × 10^6^/mL. 100 µL of cell suspension was added to a sterile flow cytometry tube and 5 µL each of CD11b and CD66b were added to each tube. The tubes were incubated on ice away from light. After incubation, a flow cytometer (NovoCyte 2060R)was used to analyze the co-expression of neutrophil surface markers, CD11b and CD66b. The number of 10,000 events within the neutrophil gate were acquired per sample for analysis. Flow cytometry detection involves three technical repetitions.

### Enzyme-linked immunoassay (ELISA)

The human histamine quantitative ELISA assay kit, human histidine ELISA assay kit, human IL-6 quantitative ELISA assay kit (MDSH2006), TNF-α quantitative ELISA assay kit (MDSH2012), and superoxide dismutase (SOD) assay kit (R&D Cat# MCRP00) were used to measure histamine, histidine, IL-6, TNFα, and SOD levels, respectively. Apoptosis detection involves three technical repetitions.

### CCK-8 cell viability assay

Cells in different groups were diluted to a final concentration of 2 × 10^6^ cells/mL and 100 µL cell suspension was added per well to a 96-well plate. The plates were cultured in an incubator for 24 h (37 °C, 5% CO_2_). 10 µL CCK8 solution (BIOMEOICAL, B1099) was added per well and the plate was incubated in an incubator for 2 h. A microplate reader was used to compare the changes in 450 nm absorbance with time between the various groups and the control group (0 mmol/L-His); 450 nm absorbance, OD450, measured by the microplate reader reflects the number of viable cells.

### Cell apoptosis assay

Apoptosis was assessed using a fluorescence-based method measured by flow cytometry. Specifically, we utilized the Annexin V-FITC Apoptosis Detection Kit (C1062S, Beyotime) according to the manufacturer’s instructions. This method employs Annexin V conjugated to FITC to detect early apoptosis and propidium iodide (PI) to detect late apoptosis in a 6-well plate were grown to a confluency of 70% in the different groups and the supernatant was collected. Trypsin was used to digest adherent Nthy-ori-3-1 cells and then resuspend in complete culture medium to obtain a cell suspension. The cell suspension and supernatant cells were added to the same 5 mL centrifuge tube. Triplicate wells (cell count ≥ 5 × 105) were set up per group. The tubes were centrifuged at 1000 g for 5 min and the supernatant was discarded. PBS was used to gently resuspend the cells. The cells were then centrifuged at 1000 gfor 5 min and the supernatant was discarded. 195 µLAnnexin V-FITC solution was added to gently resuspend the cells. 5 µL Annexin V-FITC was added and the cells were gently mixed. 10 µL of propidium iodide staining solution was added and gently mixed. Aluminum foil was used to cover the tubes and the tubes were incubated at room temperature (25 °C) away from light for 20 min. During incubation, the cells were resuspended and then placed in on ice. The tubes were immediately loaded in the flow cytometer (Agilent, NovoCyte 2060R) for testing.

### Flow cytometry detection of ROS

DCFH-DA is a non-specific fluorescent probe that can be used to detect intracellular ROS levels. DCFH-DA itself has no fluorescence, but it can freely pass through the cell membrane and be hydrolyzed into DCFH by esterase. Intracellular ROS can oxidize non-fluorescent DCFH into fluorescent DCF, so the fluorescence of DCF is detected, light intensity can reflect the level of ROS within cells. Dilute the DCFH-DA(Beijing Solarbio Science & Technology Co.Ltd tem No : CA1410) probe with RPMI-1640 culture medium at a ratio of 1:1000 to make its final concentration 10 µmol/L. Cells from each experimental group were added to the DCFH-DA solution and incubated in a 37 ° C cell incubator for 25 min, cells were collected by centrifugation. The cells were resuspended twice in serum-free culture medium to fully remove the DCFH-DA probes that had not entered the cells. The ROS levels in each experimental group were analyzed by flow cytometry. The experiment was repeated three times.

### Statistical analysis

Clinical parameters were statistically analyzed using SPSS 26.0 software. Count data are represented by frequencies and constituent ratios, and the measurement data are represented by means (SDs) and medians (IQRs). The t-test was used to compare the measurement data with normal distribution between groups. The Wilcoxonrank sumtest was used to compare the measurement data with non-normal distribution between groups. The measured data of the cell experiment results were analyzed using GraphPad Prism 8. Results are expressed as mean ± standard deviation (SD) of at least 3 independent experiments. Student’s t test was used for comparison between two groups and one-way ANOVA was used for multigroup comparison. *P* < 0.05 was statistically significant.

## Results

### The serum of HT patients exhibits the most notable variation in histidine levels

**Fig. 1:**
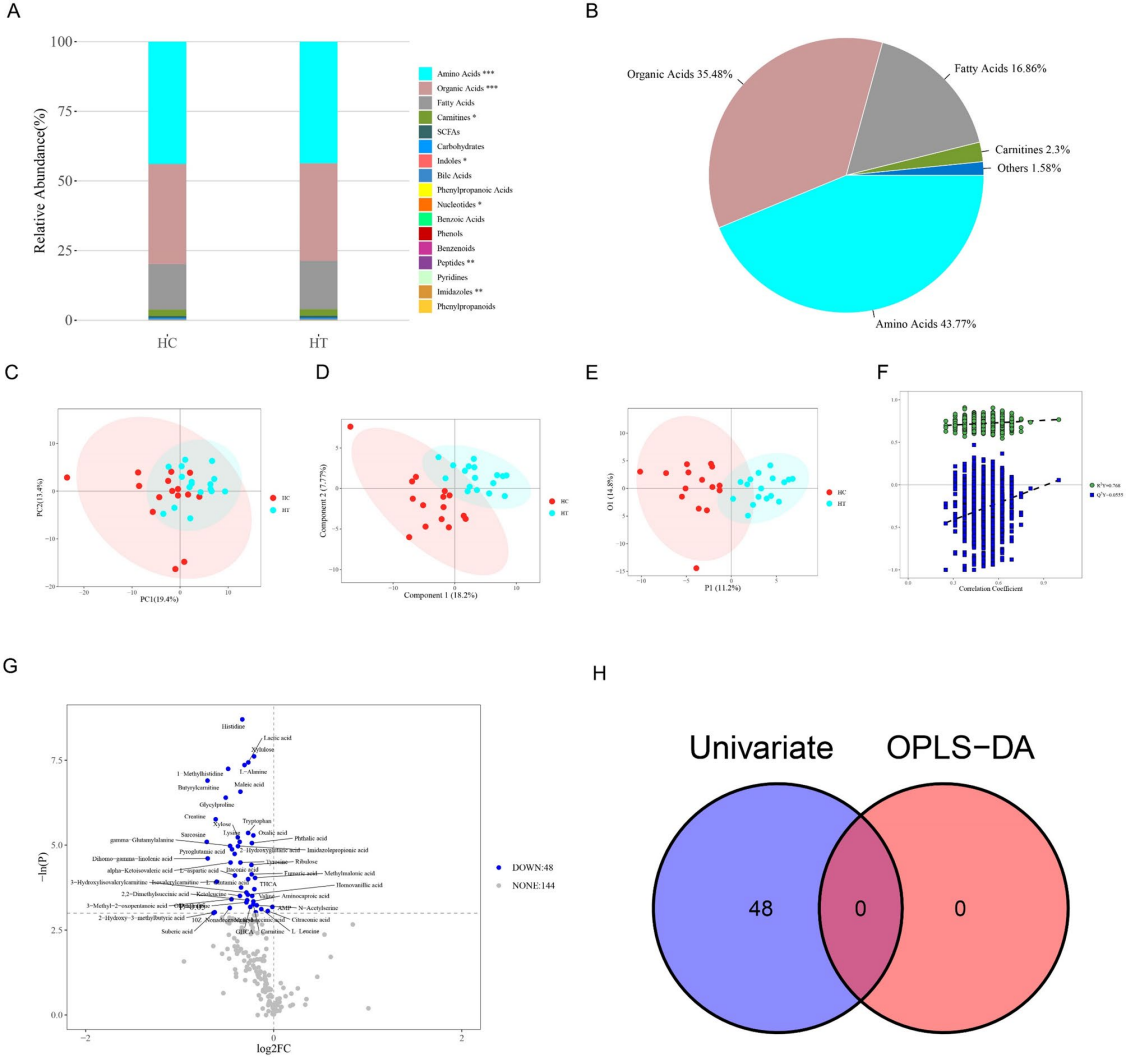
Serum metabolomics analysis of Hashimoto’s thyroiditis. (**A**) Bar charts of metabolites in Group HC and Group HT, with different colors representing different types of metabolites. (**B**) The percentage of different metabolite types in the two groups of samples. (**C**) Principal Component Analysis, PCA score graph, the horizontal axis represents the first principal component (P1), and the vertical axis represents the second principal component (P2). The distribution of each sample can be seen from the coordinate system. (**D**) Partial least squares-Discriminant Analysis, PLS-DA score plot. (**E**) Orthogonal Partial least squares-Discriminant Analysis OPLS-DA score plot. (**F**) OPLS-DA permutation test, R2Y = 0.768, Q2Y = 0.0555. (**G**) Metabolite volcano plot, obtained through unidimensional statistical analysis (Student’s t test, *p* < 0.05), screening conditions: *p* < 0.05 and the absolute value of log2FC > 0 (Fold Change FC between groups), up-regulated metabolites are represented in red, down-regulated metabolites are represented in blue. (**H**) Multi-dimensional and unidimensional Venn diagrams for metabolite analysis.

Metabolomics analysis was conducted on serum samples from 20 patients with Hashimoto’s Thyroiditis (HT) and 20 healthy control (HC) individuals using ultra-high-performance liquid chromatography-tandem mass spectrometry (UPLC-MS/MS). A total of 192 metabolites were identified, categorized into 17 classes based on their composition(Fig. 1A), including amino acids, organic acids, fatty acids, carnitine, short-chain fatty acids, carbohydrates, indoles, bile acids, phenylpropanoids, nucleotides, benzoic acids, peptides, pyridines and imidazoles. Based on the pie chart (Fig. 1B), it is evident that amino acids exhibit the highest content among the tested metabolites, leading us to hypothesize a potential association between amino acids and HT pathogenesis. Subsequently, we performed separate PCA, PLS-DA, and OPLS-DA analyses on the tested metabolites (Fig. 1CDE). The results depicted in Fig. 1CDE clearly demonstrate distinct separation between HC and HT samples, indicating differential serum metabolite profiles. Notably, our study effectively discriminates between these two groups. Furthermore, permutation test analysis of the OPLS-DA model (R2Y = 0.768, Q2Y = 0.0555) revealed its unsuitability for identifying differential metabolites in this context (Fig. 1F). To address this limitation, univariate statistical analysis was employed using screening criteria of *P* < 0.05 and |log2FC| > 0. The volcano plot (Fig. 1G) illustrates the combined results obtained from both OPLS-DA and univariate analysis while the Venn diagram (Fig. 1H) provides a visual representation of these findings. Consequently, we identified a total of 48 differential metabolites based on rigorous selection criteria. These differentially expressed metabolites were then ranked in descending order of p-value as presented in Table 3; notably histidine emerged as the most significant differential metabolite indicative of its pivotal role in distinguishing HC from HT groups.

**Table 3:** 48 distinct metabolites.

Metabolite	p value (*p* < 0.05)	log2FC
Histidine	0.000165905	−0.334264833
Xylulose	0.00049146	−0.209058569
Lactic acid	0.000587011	−0.2697629
L-Alanine	0.000632854	−0.309940885
1-Methylhistidine	0.000710409	−0.484676355
Butyrylcarnitine	0.001007678	−0.704603005
Maleic acid	0.001390148	−0.355102213
Glycylproline	0.001662923	−0.510846907
Creatine	0.003127423	−0.616260858
Tryptophan	0.004694303	−0.271999395
Oxalic acid	0.005035776	−0.216690509
Lysine	0.005350738	−0.38255568
Sarcosine	0.006085801	−0.711193796
Xylose	0.006085801	−0.361690334
Phthalic acid	0.00633429	−0.232174449
gamma-Glutamylalanine	0.00687024	−0.464006043
Imidazolepropionic acid	0.006907013	−0.378669476
Pyroglutamic acid	0.007569	−0.443555338
2-Hydroxyglutaric acid	0.008690897	−0.41481026
Dihomo-gamma-linolenic acid	0.009972716	−0.702570904
Tyrosine	0.011226259	−0.35552684

Subsequently, we established in vitro cell models of NETs to further explore the pathogenesis of HT related to histidine.

### High iodine concentration induces increase in neutrophil NETs synthesis and decreases histidine

**Fig. 2:**
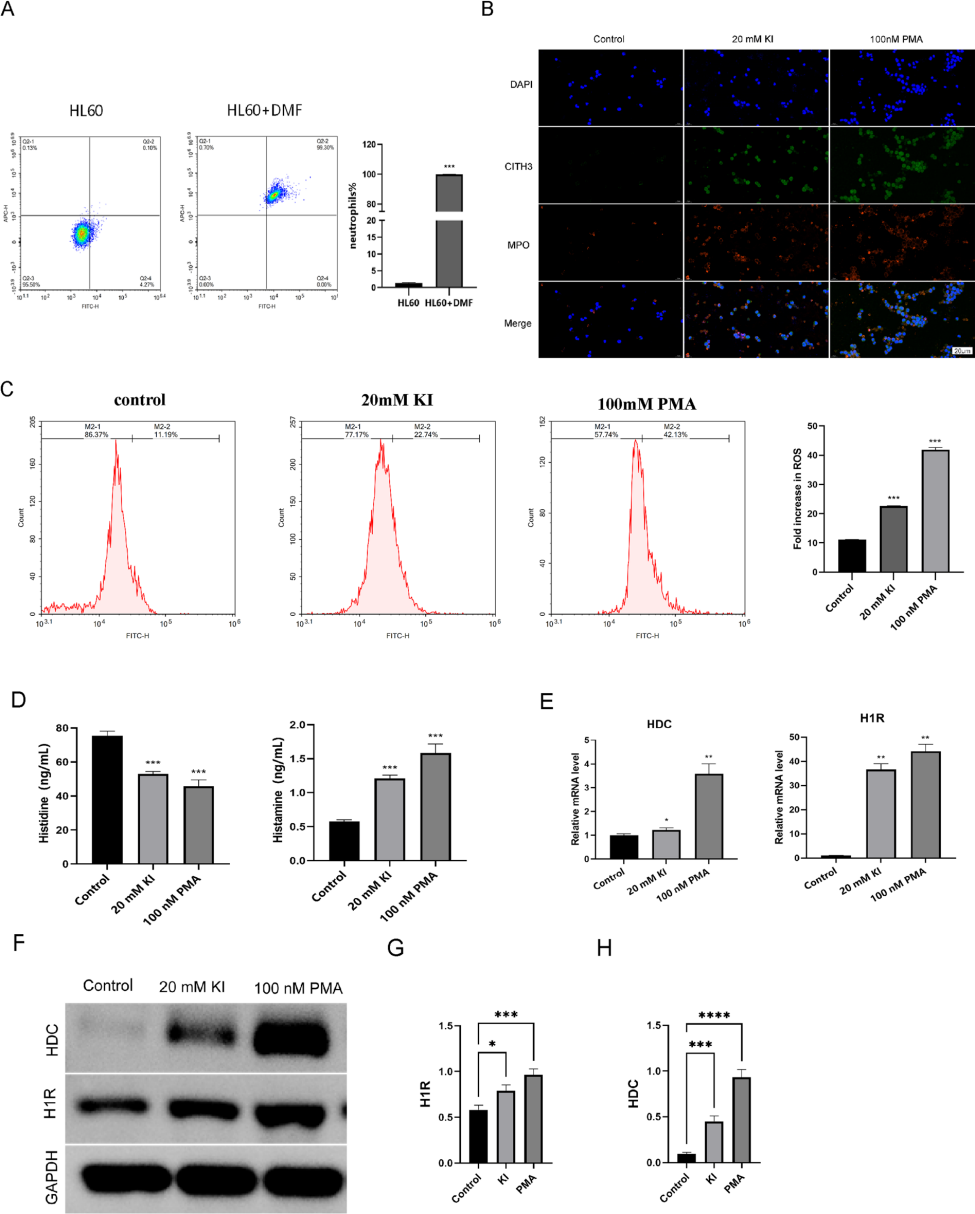
High-concentration iodine promotes the formation of NETs by neutrophils. (**A**) DMF induces the differentiation of HL60 into neutrophils. The horizontal vertical axis represents FITC-CD66b, the vertical axis represents APC-CD11b, and Q2-2 represents the double positive rates of CD11 + and CD66b+. (**B**) 20mM KI can induce neutrophils to generate NETs, which can be used to construct NETs models. DAPI shows blue fluorescence and marks nucleic acids, while CITH3 emits green fluorescence and marks NETs. MPO emits red fluorescence to mark NETs. (**C**) As the generation of NETs increases, ROS also increases. (**D**) With the increase of NETs generation, histamine increased and histidine decreased. (**E**) With the increase of NETs, the expression of both HDC and H1R genes was detected to increase by qPCR. (**F**) NETs increased, and Western Blot detected that the protein expressions of HDC and H1R both increased. *P < 0.05, **P < 0.01, ***P < 0.001.

Next, we used a NETs cell model to further study the relationship between serum metabolites and HT. Aneta Manda-Handzlik et al.^25^ found that when HL-60 cells were differentiated with all-trans retinoic acid (ATRA), dimethyl sulfoxide (DMSO), or dimethylformamide (DMF) respectively, the results showed that DMF was the best stimulant for HL-60 cell differentiation in NETs studies. It can most effectively differentiate HL-60 cells into granulocytoid cells capable of releasing NETs. Therefore, we used DMF to induce HL60 cells to differentiate into neutrophils (CD11b+/CD66b+).After 5 consecutive days of DMF stimulation, HL60 cells were analyzed by flow cytometry. We found that the frequency of CD11b+CD66b+ cells significantly increased (Fig. 2A, *P* < 0.01), showing that neutrophils were induced and could be used for subsequent experiments. In this study, we termed HL60 cells that were successfully induced by DMF (CD11b+CD66b+) as neutrophils.

To show that high iodine concentration can induce neutrophils to synthesize NETs, and to construct the NETs model, we used 20 mM KI to stimulate neutrophils. The classical inducer PMA (100 nM) was used as the positive control. Compared with the control group, immunofluorescence showed that NETs are increased in the KI group and PMA group (Fig. 2B). Concomitantly, ROS levels increased significantly (Fig. 2C). This shows that high concentration of iodine can induce NET and ROS synthesis by neutrophils.

To study changes in histidine, we used ELISA and found that histidine significantly decreased in the KI and PMA groups (Fig. 2D). As HDC, H1R, and histamine can participate in NET formation, we also measured histamine level and HDC and H1R expression (Figs. 2E-H). We found that KI and PMA induced increase histamine synthesis compared with the control group. At the same time, HDC and H1R expression increased, and their mRNA were significantly upregulated. In summary, histidine decreased in high iodine concentration-induced neutrophils and this was consistent with the above metabolomics results. This shows that histidine, histamine, HDC, and H1R are associated with.NETs.

### Histidine downregulates NETs to protect neutrophils

**Fig. 3:**
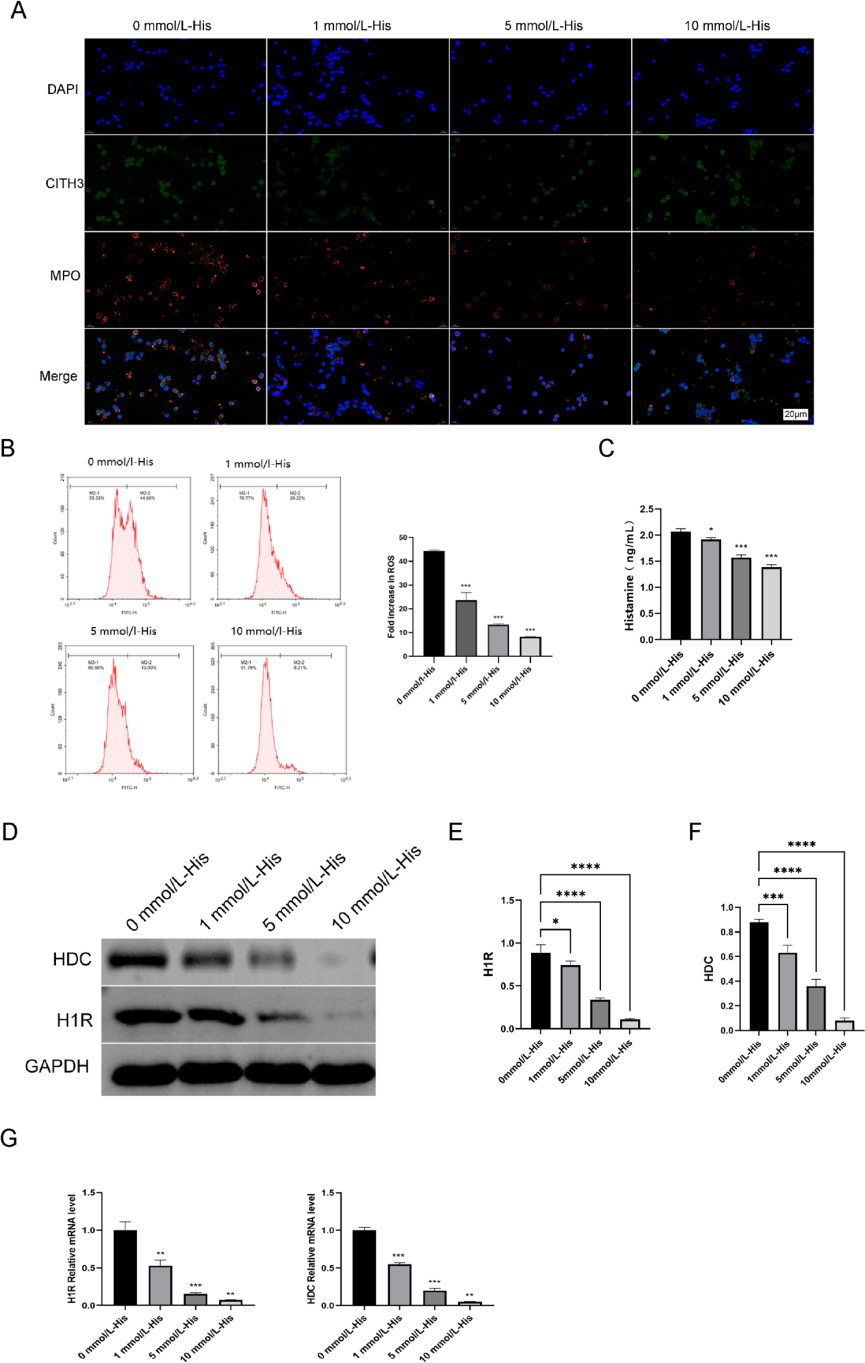
High-concentration histidine inhibits the formation of NETs. (**A**) Neutrophils induced by 20mM KI were treated with0, 1, 5, 10 mmol/L-His for 24 h respectively. The generation of NETs in each group was detected by immunofluorescence. An increase in histidine concentration led to a decrease in the generation of NETs. DAPI shows blue fluorescence and marks nucleic acids. CITH3 emits green fluorescence to label NETs. MPO emits red fluorescence to mark NETs. (**B**) Flow cytometry detection of ROS showed that an increase in histidine concentration led to a decrease in ROS. (**C**) The histamine concentration was detected by ELISA. When the histidine concentration increased, the histamine concentration decreased. (**D**–**F**) Western Blot was used to detect the protein expression of HDC and H1R. When the histidine concentration increased, the expression of HDC and H1R decreased. (**G**) qPCR was used to detect the expression of HDC and H1R genes. An increase in histidine concentration enhanced the expression of HDC and H1R genes. *P < 0.05,**P < 0.01, **P < 0.001.

Based on the above experiments, 20 mM KI can stimulate neutrophils to synthesize NETs and be used as a cell model to study HT (HT-NETs model). This model was used for subsequent studies. Next, we used different histidine concentrations (0, 1, 5, and 10 mmol/L-His) in the HT-NETs model. Absence of histidine (0 mmol/L-His) was the control group. Histidine concentration gradually increased in the remaining 3 groups and this experiment was used to examine the effects of histidine on the HT-NETs model. Histidine inhibits NETs(Fig. 3A) and exerts antioxidant and protective effects on neutrophils(Fig. 3B).This shows that histidine can inhibit NET synthesis, and has antioxidant and protective effects on neutrophils. To determine the effects of histidine on the HT-NETs model, we measured the expression of histamine, HDC, and H1R. Compared with the control group, histamine levels decreased after histidine treatment (Fig. 3C). Western blot showed that the expression of HDC and H1R were downregulated in a dose-dependent manner (Fig. 3D-G). Among the 4 groups, 10 mmol/L histidine had the most significant effects on the HT-NETs model. Overall, histidine can affect NETs, ROS, histamine, HDC, and H1R expression and these are associated with histidine concentration.

### Histidine inhibits the expression of pro-inflammatory cytokines in HT cells

**Fig. 4:**
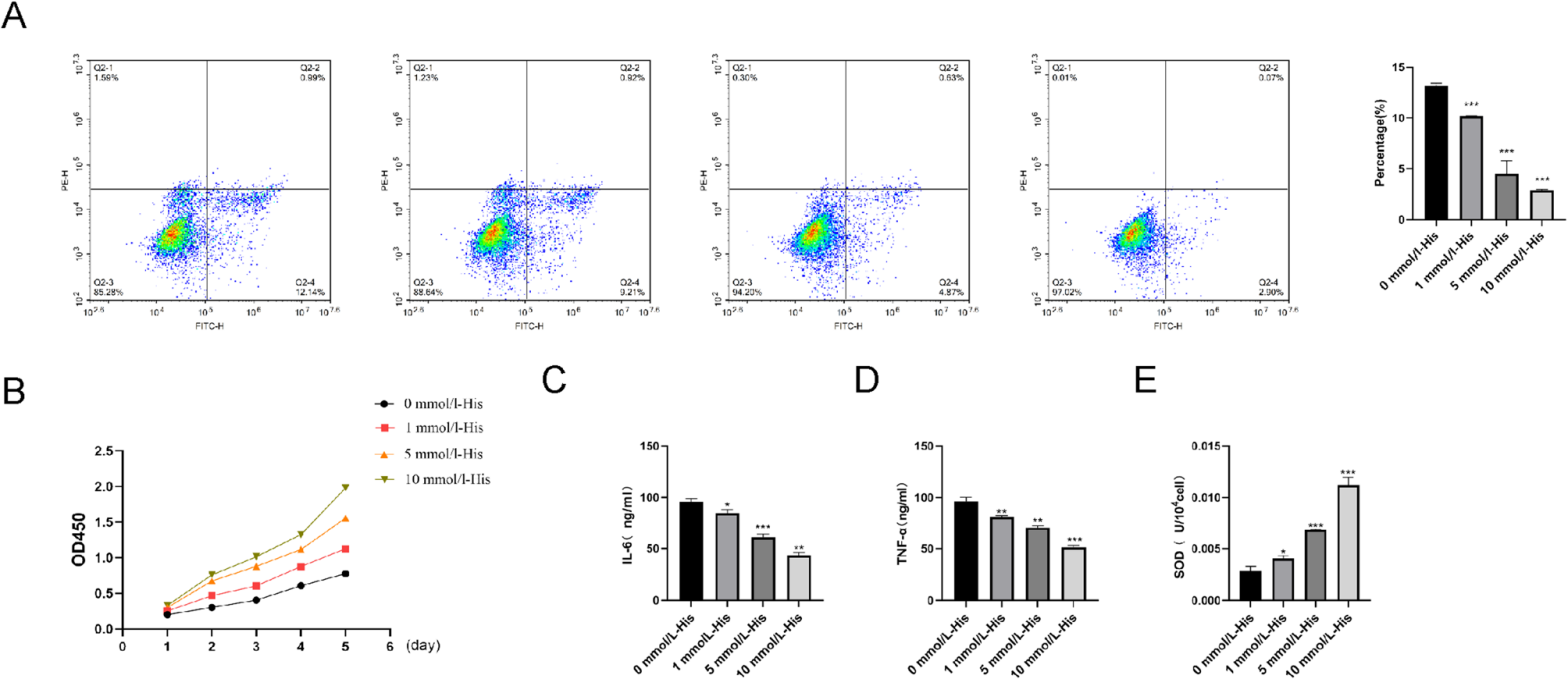
High-concentration histidine inhibits inflammation of thyroid follicular cells. (**A**) NETs treated with different concentrations of histidine (0, 1, 5, 10 mmol/L-His) were co-cultured with thyroid follicular cells. The apoptosis of thyroid follicular cells was detected by flow cytometry. The higher the histidine concentration, the less the apoptosis of cells. (**B**) CCK8 was used to detect the effect of different concentrations of histidine (0, 1, 5, 10 mmol/L-His) on the proliferation ability of thyroid follicular cells. An increase in histidine concentration led to an increase in cell viability. (**C**–**E**) ELISA was used to detect the production of TNF-α, IL-6 and SOD in thyroid follicular cells. The concentration of histidine increased, TNF-α and IL-6 decreased, and SOD increased. *P < 0.05,**P < 0.01, **P < 0.001.

To determine the mechanisms of action of histidine in HT, we co-cultured histidine-treated HT-NETs cells with normal thyroid follicular cells (Nthy-ori3-1). The histidine concentrations used in the experiment were 0, 1, 5, and 10 mmol/L-His, of which the 0 mmol/L-His was the control group. Compared with the control group, apoptosis of thyroid follicular cells decreased in a dose-dependent manner (Fig. 4A). Using a CCK-8 assay, we found that cell viability increased gradually with histidine concentration (Fig. 4B). This shows that histidine can inhibit apoptosis and increase viability of thyroid follicular cells and has protective effect. Hence, it could ameliorate damage to thyroid follicular cells. Next, we determined expression levels of pro-inflammatory cytokines TNFα and IL-6 and the antioxidant enzyme SOD by ELISA. We found that TNFα and IL-6 were significantly decreased, and SOD was significantly increased compared with the control group, and showed histidine concentration-related fluctuations (Fig. 4C-E). These results showed that histidine can inhibit the expression of pro-inflammatory cytokines TNFα and IL-6 and increase the expression of the antioxidant enzyme SOD. Hence, it has anti-inflammatory and antioxidant effects.

### Histidine play an anti-inflammatory role by reducing NF-κB activation

**Fig. 5:**
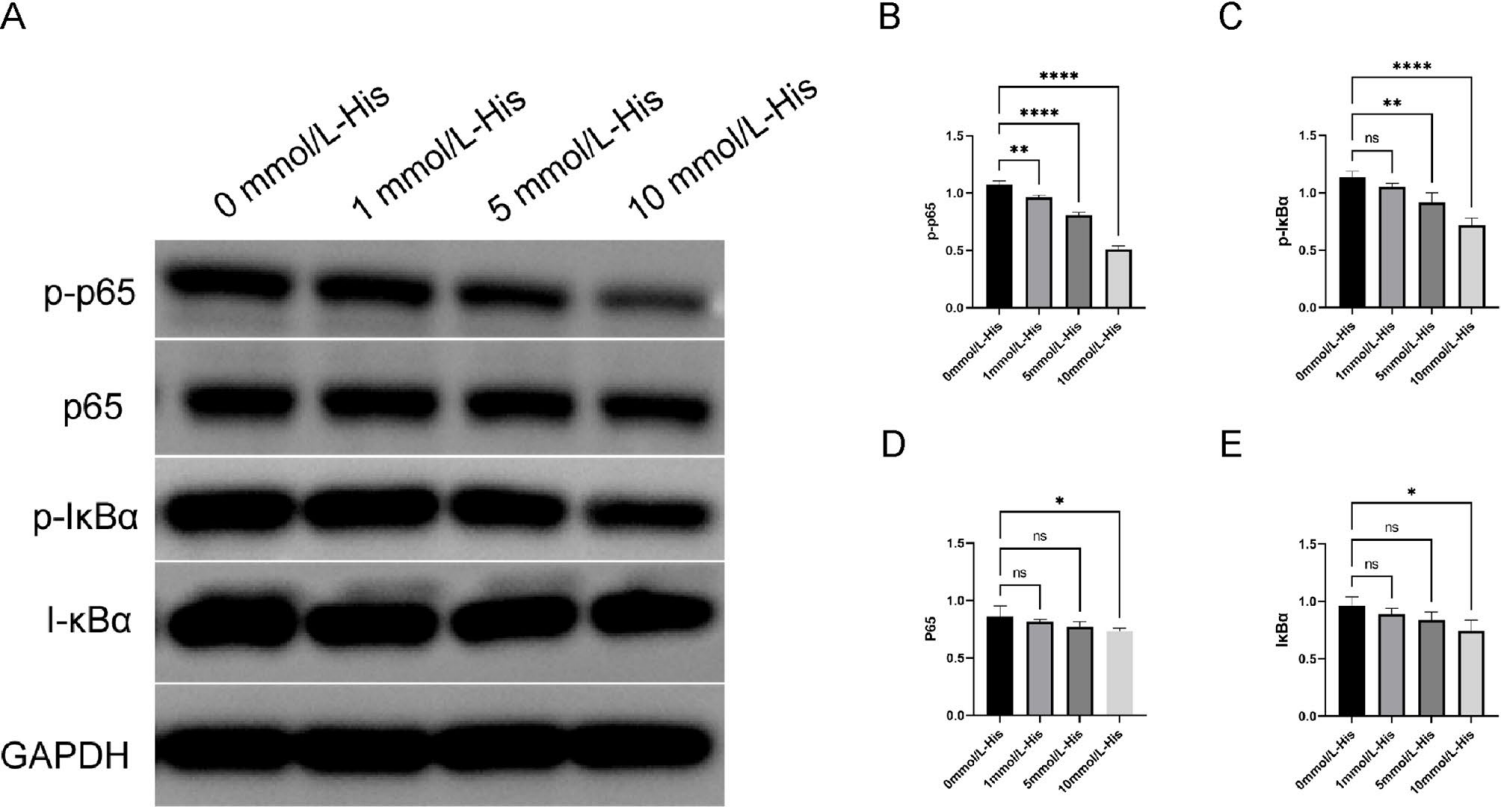
High-concentration histidine inhibits inflammation of thyroid follicular cells by suppressing the NF-κB signaling pathway. (**A**–**E**) Western Blot was used to detect the expression of NF-kB pathway proteins (p-P65, p65, p-κBα, IκBα) in histidine concentrations (0, 1, 5, 10 mmol/L-His) in each group. The histidine concentration increased. The protein expressions of p-P65, p65, p-κBα, and IκBα decreased. *P < 0.05,**P < 0.01, **P < 0.001, NS, notsignificant.

we tested the hypothesis that the NF-κB signaling pathway may participate in the pathogenesis of HT. Western blot was used to measure the expression of NF-κB signaling pathway-related proteins IκBα, p-IκBα, p65, and p-p65. The 0 mmol/L-His was used as the control group. The experiment showed that compared with the control group, p-IκBα and p-p65 expression in thyroid follicular cells decreased in presence of histidine and the inhibitory effects of high histidine concentration (10 mmol/L-His) on p-IκBα and p-p65 were more significant (Figs. 5A-C). The changes in p65 and IκBα in the low histidine concentration groups (1 and 5 mmol/L-His) were not significant (*P* > 0.05) and p65 and IκBα experiment decreased only in the 10 mmol/L-His group (Figs. 5A, DE). Thus, our results suggest that histidine may ameliorate thyroid follicular cell inflammation through the NF-κB pathway. In summary, our results showed that histidine can inhibit the expression of HT pro-inflammatory cytokines through the NF-κB signaling pathway to ameliorate thyroid follicular cell inflammation.

## Discussion

Histidine is an essential amino acid in humans. Previous studies showed that it has antioxidant and anti-inflammatory effects and can ameliorate oxidative stress and inflammation in a rat model of diabetes type 2^26^. Studies have shown that minerals such as selenium, zinc, vitamins D and C, as well as probiotics, can be used as adjunctive treatments to control oxidative damage and poor prognosis of HT^27^. Similarly, histidine has antioxidant stress effects and can also be used as an adjunctive treatment for HT. There are few studies on the protective effects of histidine in HT. We carried out quantitative analysis of serum metabolites and found that histidine was significantly decreased in HT patients. Moreover, high iodine concentration treated mice are often used as an animal model for HT studies. Our group previously used high KI concentration to stimulate neutrophils and construct a cellular model to study the relationship between NETs and HT. Here, we showed that histidine decreased the synthesis of ROS and NETs in the HT-NETs model, while it increased HDC, H1R, and histamine. It is widely known that ROS is a critical marker of oxidative damage as it can disrupt nuclear and cell membranes and mediate NET production^28^. In addition, using co-culture of the HT-NETs model and Nthy-ori3-1 cells, we showed that histidine inhibits the expression of pro-inflammatory cytokines TNFα and IL-6 and increased antioxidant enzyme SOD synthesis in Nthy-ori3-1 cells through suppression of the NF-κB pathway.

Our results were consistent with other studies. Histidine is decreased in inflammatory diseases such as Takayasu arteritis, systemic lupus erythematosus, and rheumatoid arthritis^29–31^. In the cell co-culture experiment, the expression of pro-inflammatory cytokines was increased, and antioxidants decreased in absence of histidine (0 mmol/L-His group). Many stimuli, such as microorganisms and its products, cytokines, immune complexes, autoantibodies, and chemicals can induce neutrophils to release NETs. It has also been reported that LPS could induce NETs synthesis in a mouse model and activated neutrophils produce large amounts of pro-inflammatory cytokines, thereby mediating lung injury^32^. This was similar to our study in which KI-induced neutrophils was used to construct a HT-NETs model and the experimental results were identical.This is related to the construction of neutrophils induced by KI.The changes in the NETs model are similar, and the experimental results are similar, except that we use the thyroiditis correlation.The results can better explain the role of neutrophils in the pathogenesis of thyroiditis.

Multiple studies have demonstrated that histidine has anti-inflammatory and antioxidant effects, but there are relatively few studies on whether high levels of histidine can alleviate HT.In the cell co-culture experiment, histidine increased, the expression of pro-inflammatory cytokines (TNF-α, IL-6) decreased, and the expression of antioxidant enzyme (SOD) increased. We found that histidine has anti-inflammatory and antioxidant effects in HT thyroid follicular cells. This is in agreement with previous studies showing that histidine has anti-inflammatory and antioxidant effects in other diseases. First, a study showed that histidine supplementation can improve lung function, decrease inflammatory cells and cytokines in bronchoalveolar lavage in COPD mice^33^. Second, a study found that histidine supplementation could inhibit liver oxidative stress induced by high salt exposure^34^. In addition, histidine deficiency could inhibit antioxidant capacity in largemouth bass intestines and induce gut endoplasmic reticulum stress, inflammatory responses, and apoptosis^35^. Herein, we showed that histidine inhibits ROS and NETs synthesis to exert its anti-inflammatory effects. A study found that imidazole in the molecular structure of histidine can scavenge ROS produced during the acute inflammatory responses, thereby exerting protective effects on inflamed tissues.

As for the specific mechanism of exogenous histidine playing the above role, we have also explored. In this study, we found that histidine treatment can inhibit the expression of several members of the NF-κB signaling pathway. It is well known that the NF-κB signaling pathway controls important biological functions such as immune and inflammatory responses, differentiation, cell growth, tumorigenesis, and apoptosis^36–38^. Other studies found that histidine could inhibit the expression of pro-inflammatory cytokines in adipocytes through the NF-kB pathway^39^. Interestingly, a study showed that the NF-kB pathway is intimately associated with thyroid cell survival and expression of key thyroid markers, such as thyroid peroxidase, and thyroglobulin, and is an essential transcription factor for maintenance of thyroid function^40^. Therefore, we hypothesized that NF-κB participates in HT occurrence and this was experimentally validated. In the co-culture experiment of NETs and thyroid follicular cells, exogenous histidine can inhibit NF-κB signaling pathway, reduce the expression of pro-inflammatory cytokines (TNF-α, IL-6), and increase the expression of antioxidant enzyme (SOD). In short, histidine ameliorates thyroid follicular cell inflammation through the NF-κB signaling pathway. Of course, the NF-κ b pathway in this article is merely a preliminary mechanism exploration, initially suggesting that this pathway may be involved in mediating the anti-inflammatory effect of histidine. However, it has some shortcomings. No functional recovery experiments have been conducted to directly confirm that the NF-κB pathway is a necessary condition for histidine to exert its function, and there is a lack of evidence of specific activation of the pathway. If NF-κ B-specific activators can be used simultaneously with histidine treatment and it is demonstrated that this operation can completely or partially block the anti-inflammatory effect of histidine, it will provide the most direct causal evidence for the mechanism. This limitation is also the primary direction of future research.

Interestingly, our study results also found that HDC, H1R, and histamine changes with histidine concentration in a dose-dependent manner. Previous studies found that HDC catalyzes histamine synthesis from L-histidine and participates in allergic reactions, gastric acid secretion, immunomodulation, and development of hematopoietic stem cells^41^. Previous studies have shown that HDC, H1R, and histamine are commonly involved in allergic reactions.Histamine can bind to H1R and this mediates allergic reactions in normal situations^42^. Studies have shown that IL-33 can directly or indirectly (via IL-5) induce HDC in a variety of cells, especially in c-kit+ cells and mature mast cells^43^. It has also been shown that the HDC GC box within the proximal enhancer of the mouse and human HDC genes is critical for Hdc gene transcription, histamine synthesis, and histamine-mediated allergic reactions in vitro and in vivo^44^. In addition, studies have shown that HDC expression can be regulated by the transcription factor FLI1 (Friend leukemia virus insertion site 1) to control inflammatory signaling and leukemia progression^45^. Interestingly, studies have shown that a possible mechanism for the regulation of HDC activity is the change of histidine itself or the concentration of various compounds that affect HDC activity. For example, histidine metabolite α-methyl-L-histidine is a strong specific inhibitor of HDC^46^. Therefore, the exogenous histidine concentration increased, the HDC activity was significantly inhibited, and the expression of HDC and H1R decreased. The relationship between histidine and HDC needs to be further studied.

Apart from histidine, metabolites may also be involved in the pathogenesis of HT. the direct correlation between organic acids and HT is rare, but some organic acids have anti-inflammatory effects in other diseases. For instance, creatine supplementation can alleviate post-exercise fatigue through anti-inflammatory effects in skeletal muscles and the brain^47^, and fumaric acid inhibits inflammation by regulating inflammatory and oxidative stress biomarkers in arthritis rats^48^. In addition, some fatty acids are also associated with inflammation. Studies have shown that HT can affect fatty acid metabolism in thyroid tissue^49^. Sarandi et al. conducted metabolomics analysis on the plasma of patients with Hashimoto’s thyroiditis and found that Hashimoto’s thyroiditis is related to fatty acid metabolic dysfunction^50^, some people have also found the effect of a high-fat diet on thyroid autoimmunity in female rats^51^. Tyrosine has a certain connection with HT. Tyrosine is the most important amino acid involved in the synthesis of thyroid hormones^52^. Low levels of tyrosine can cause problems such as hypothyroidism, hypotension, chronic fatigue and slow metabolism^53^. Studies have shown that excessive iodine intake can lead to Hashimoto’s thyroiditis^54^, and long-term exposure to elevated iodine levels in drinking water is associated with the occurrence of autoimmune thyroid diseases in adults^55^. At present, there is no evidence suggesting that histidine can directly affect thyroid antibodies, and further research is needed.

Regrettably, due to time and condition constraints, the sample size we used for the experiment was only 20 cases. However, this did not affect the accuracy of the experiment. For example, Tao Luo et al. conducted serum metabolome analyses on 14 HT TPOAb-positive patients, 4 TGAB-positive patients and 14 gender-matched healthy controls, and discovered the differences in metabolites among them^56^. Similarly, Boshen Gong et al. evaluated the composition and metabolic characteristics of the gut microbiome in 23 HT patients and 25 healthy individuals, and ultimately found that iodine intake alters the composition and metabolic changes of the gut microbiome, affecting the microbiota-gut-thyroid axis^57^. Of course, in the future, we will try our best to increase the sample size to ensure that the experiment is more accurate. Furthermore, if there were negative or positive controls for immunofluorescence in this study, the experimental conclusion might be more persuasive.

## Conclusions

**Fig. 6:**
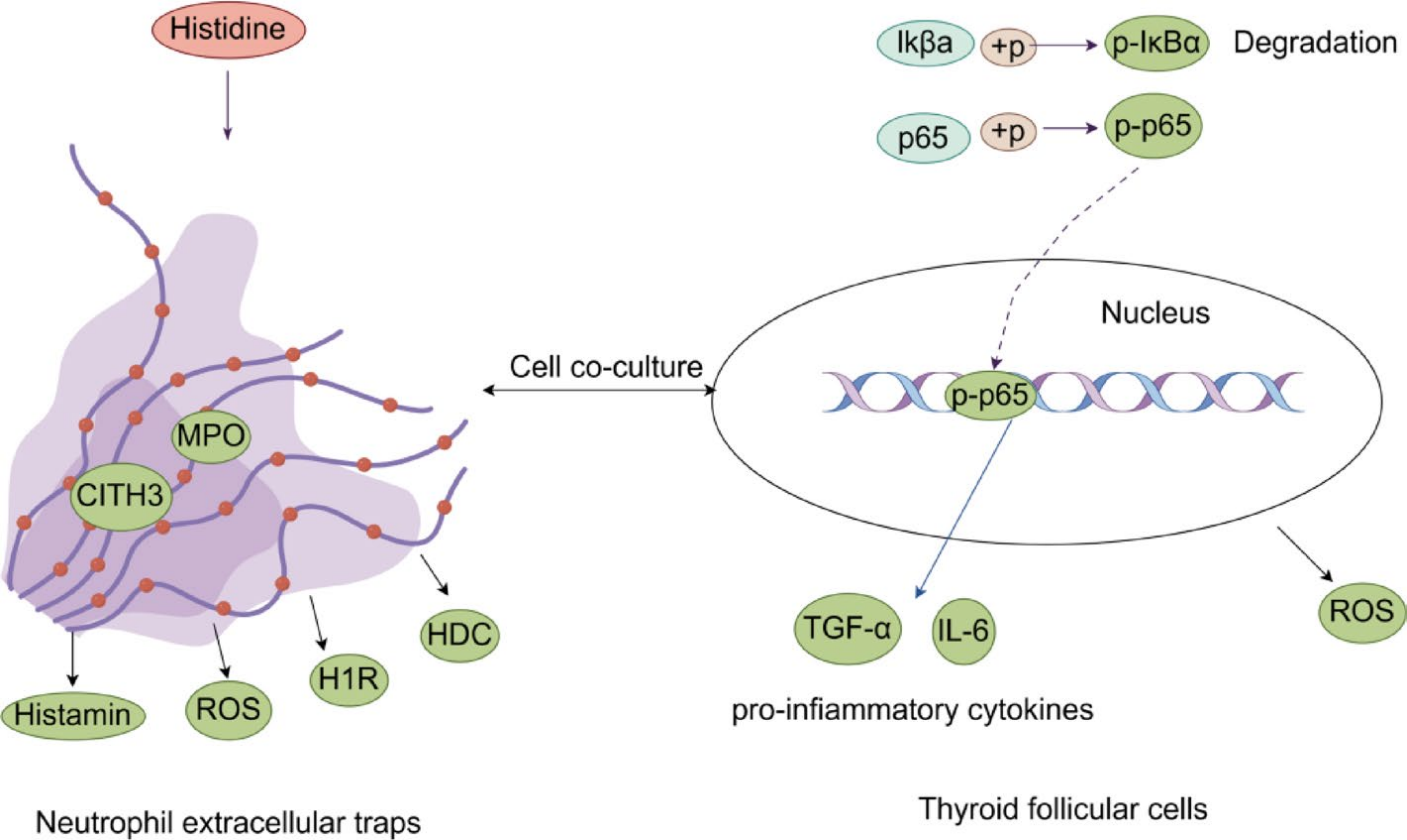
Histidine ameliorates thyroid follicular cell inflammation through modulation of the NETs-NF-κB pathway. High concentrations of histidine inhibit the generation of NETs in neutrophils, leading to an increase in ROS, histamine, H1R, and HDC. This results in the inhibition of the NF-κB signaling pathway in thyroid follicular cells, thereby reducing TNF-α and IL-6(Red indicates an increase and green indicates a decrease).

Overall, histidine improves HT through the NETs-NF-κB pathway (Fig. 6). Our metabolome analysis showed that histidine is an important metabolite in the pathophysiology of HT. We showed that histidine has protective effects in high KI concentration-induced neutrophils; inhibits NETs synthesis and expression of pro-inflammatory cytokines in thyroid follicular cells, ameliorating thyroid follicular cell inflammation. Histidine effects were mainly achieved through suppression of the NF-κB signaling pathway. In summary, Our research highlights the protective effect of histidine in HT and is expected to become a new type of drug for treating HT patients.

## Supplementary Information

Below is the link to the electronic supplementary material.

Supplementary Material 1

Supplementary Material 2

Supplementary Material 3

## Data Availability

The datasets used and/or analyzed in the current study are available from the corresponding authors upon reasonable request. The raw sequencing data has been uploaded to the MetaboLights database. The original database link is: https://www.ebi.ac.uk/metabolights/editor/MTBLS12333/descriptors.
