# Supplementary figures and images for: Histidine alleviates Hashimoto’s thyroiditis via the neutrophil extracellular traps-NF-κB signaling pathway
Source: Sci Rep. 2026 Mar 26;16:15148. doi: 10.1038/s41598-026-45671-2 (PMC13172347; doi:10.1038/s41598-026-45671-2)

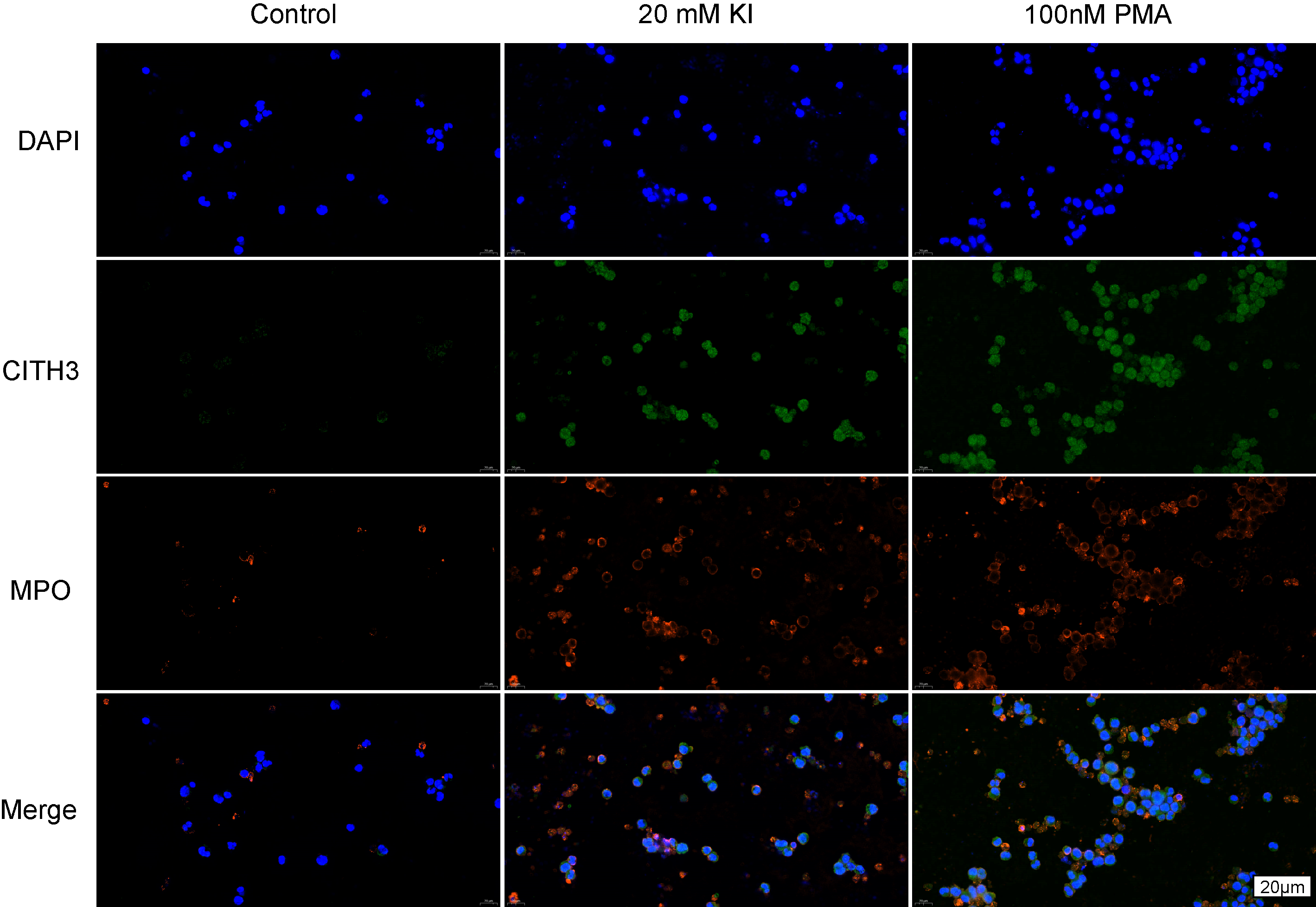

Supplement: Supplementary file 1 — Supplementary Material 1 [file 41598_2026_45671_MOESM1_ESM.zip › NETs/Figure 2B.tif]

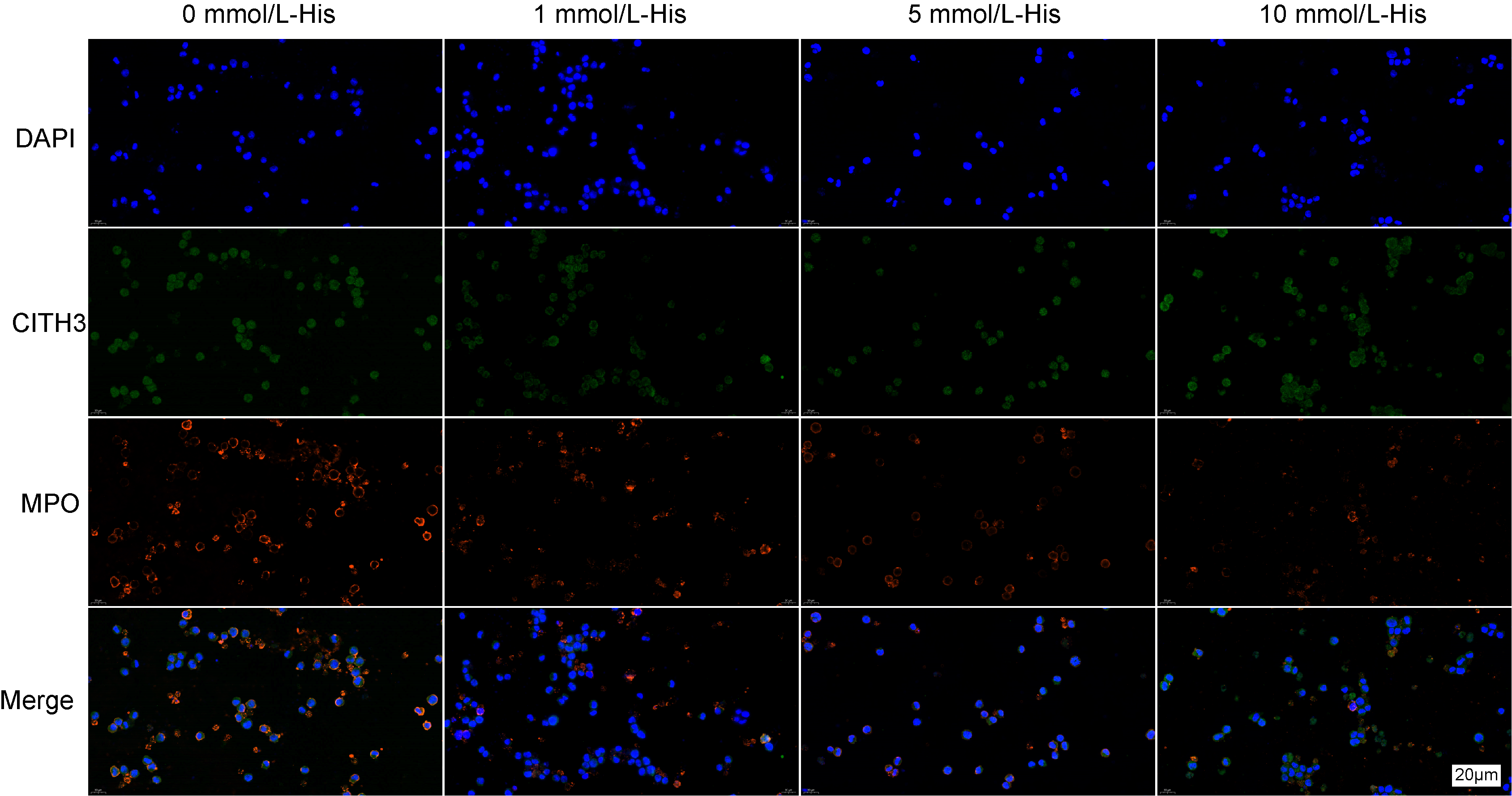

Supplement: Supplementary file 1 — Supplementary Material 1 [file 41598_2026_45671_MOESM1_ESM.zip › NETs/Figure 3A.tif]

Supplement2:

Figure 2F

HDC

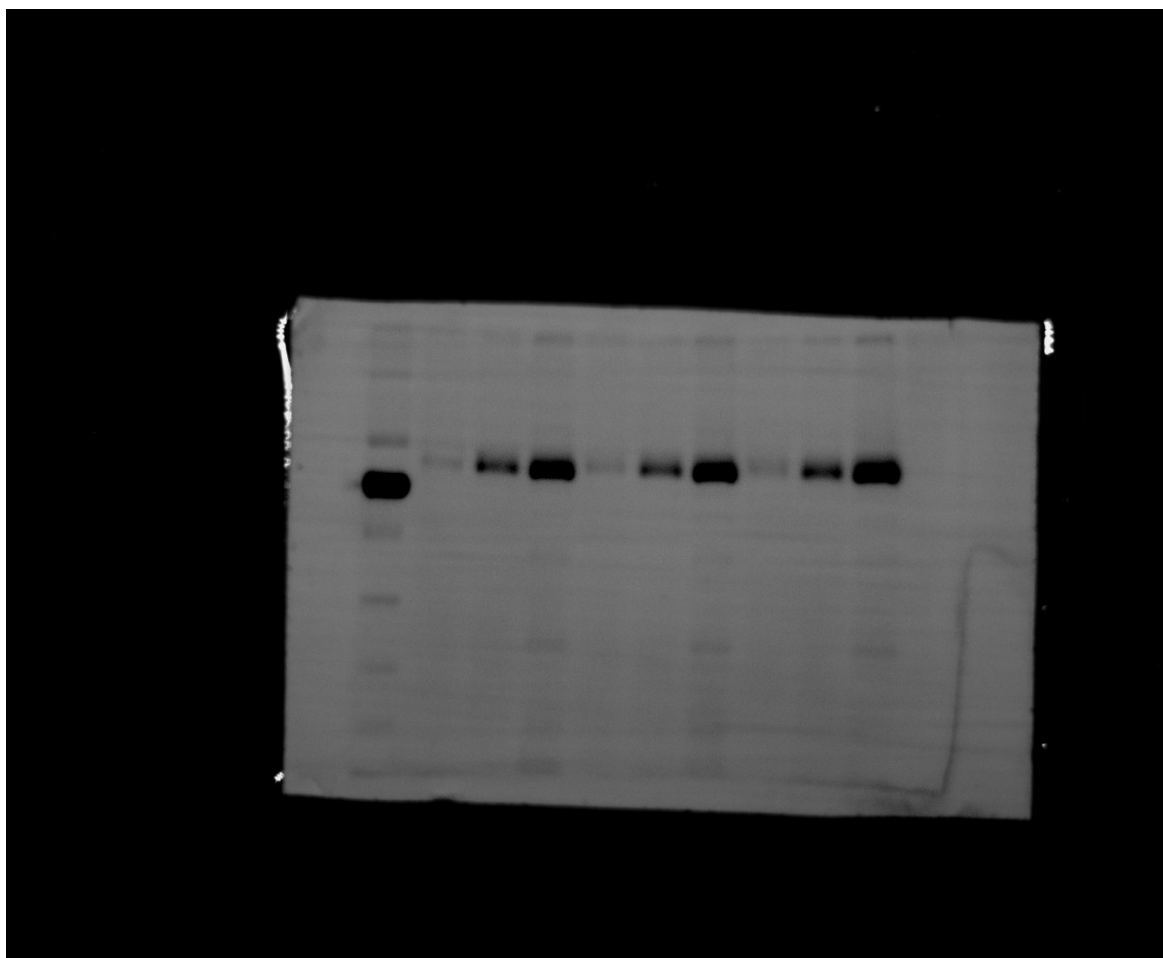

H1R

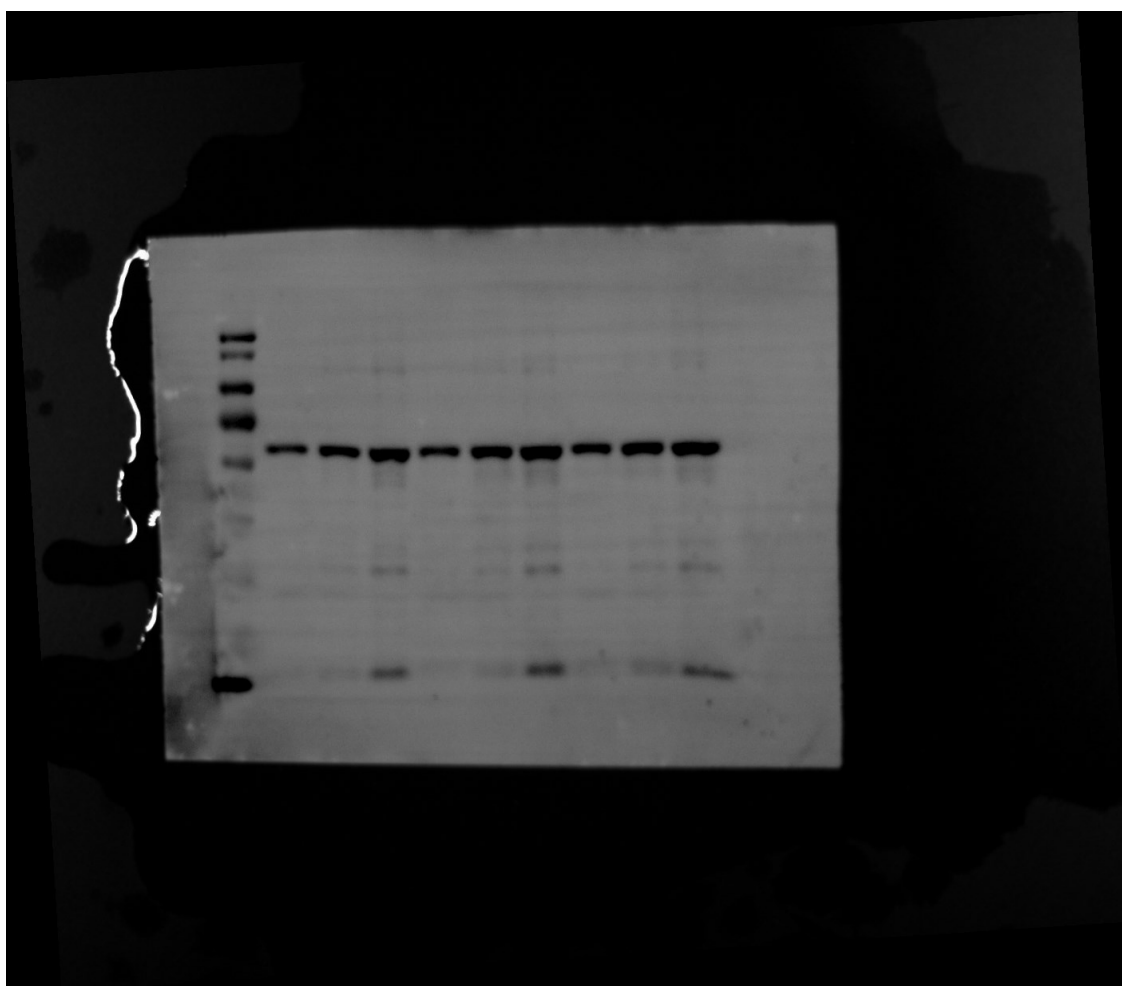

GAPDH

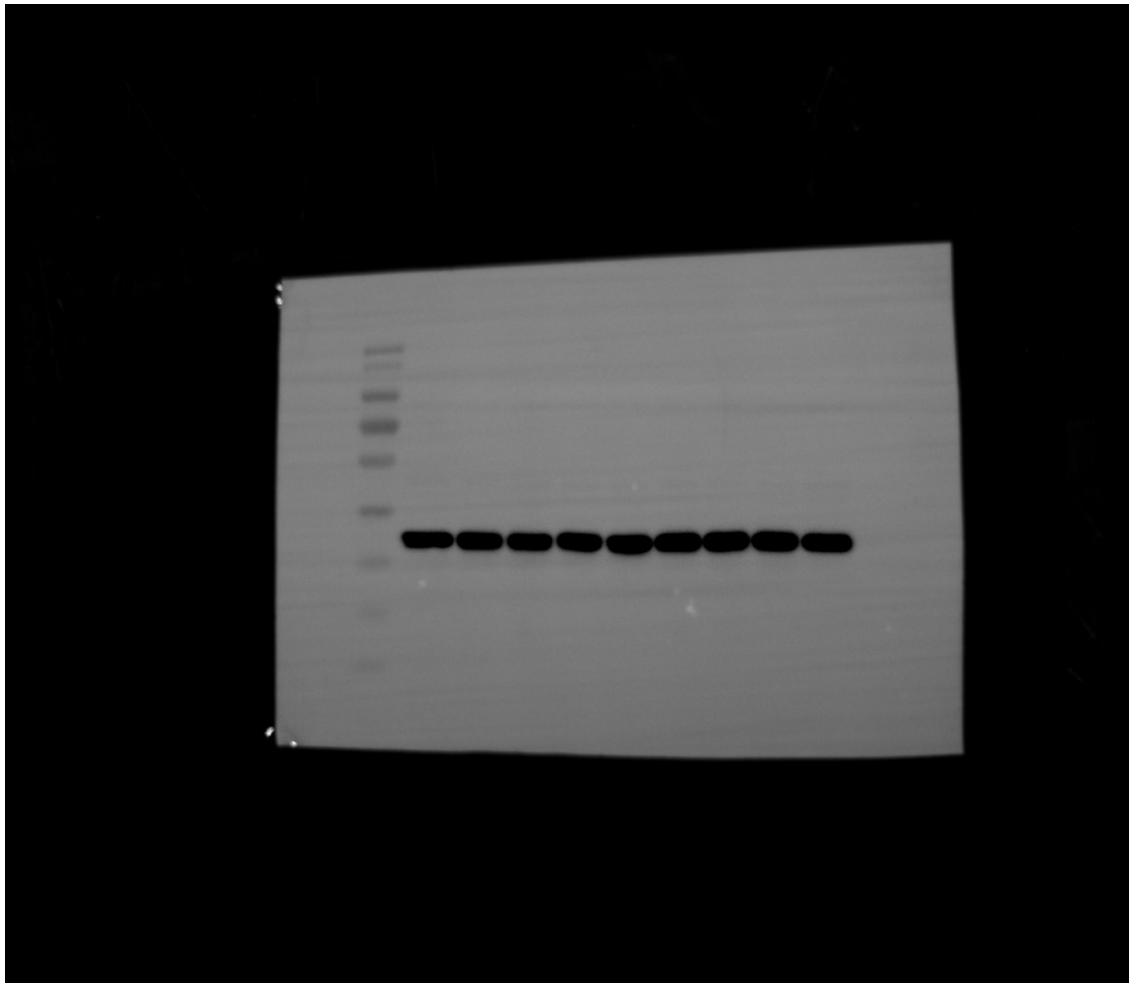

Figure 3D

HDC

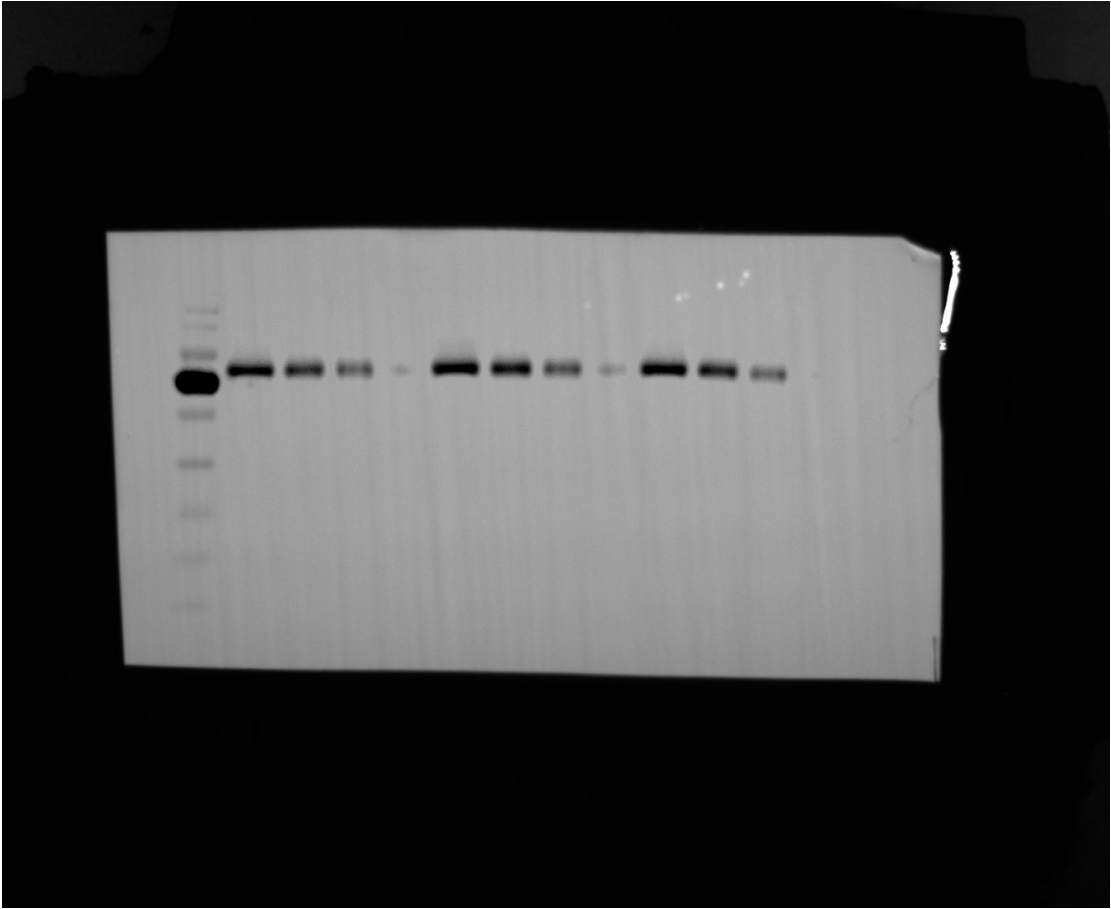

H1R

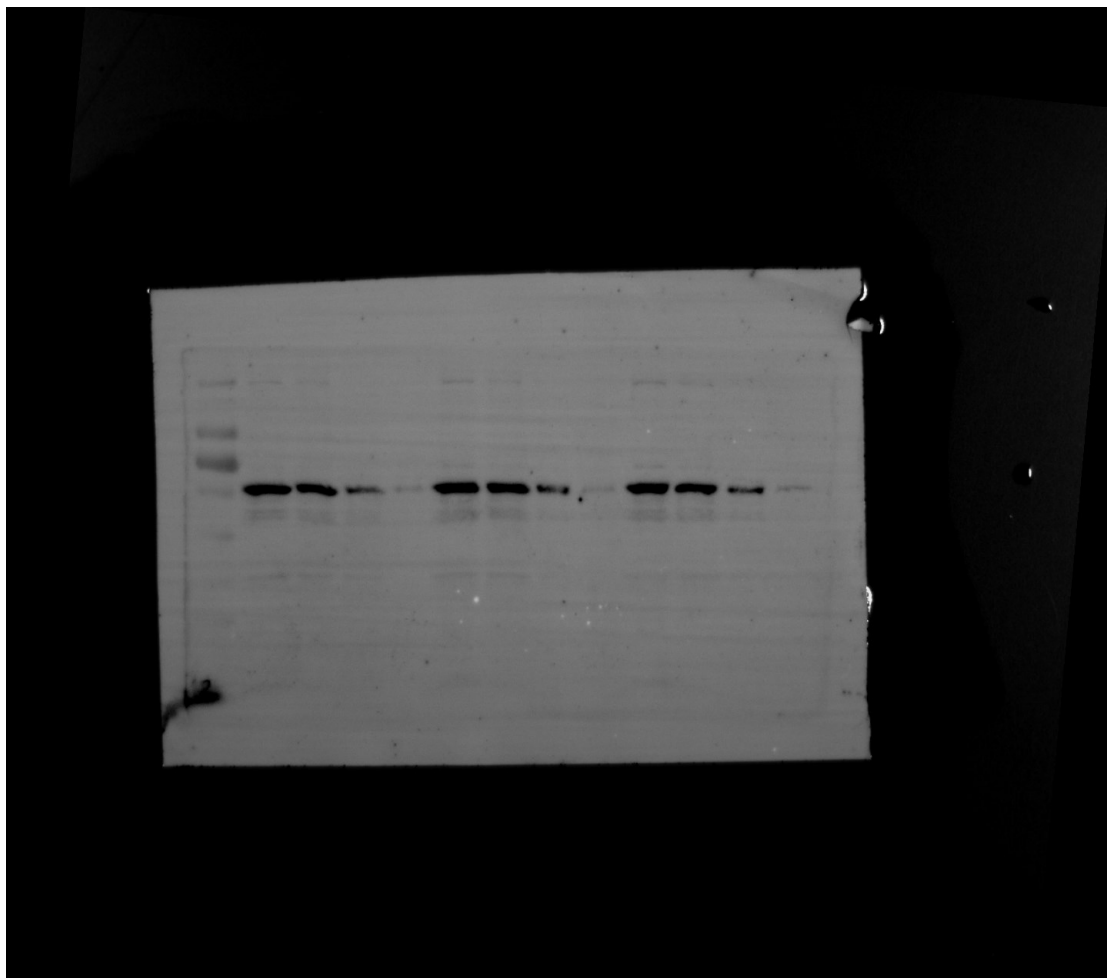

GAPDH

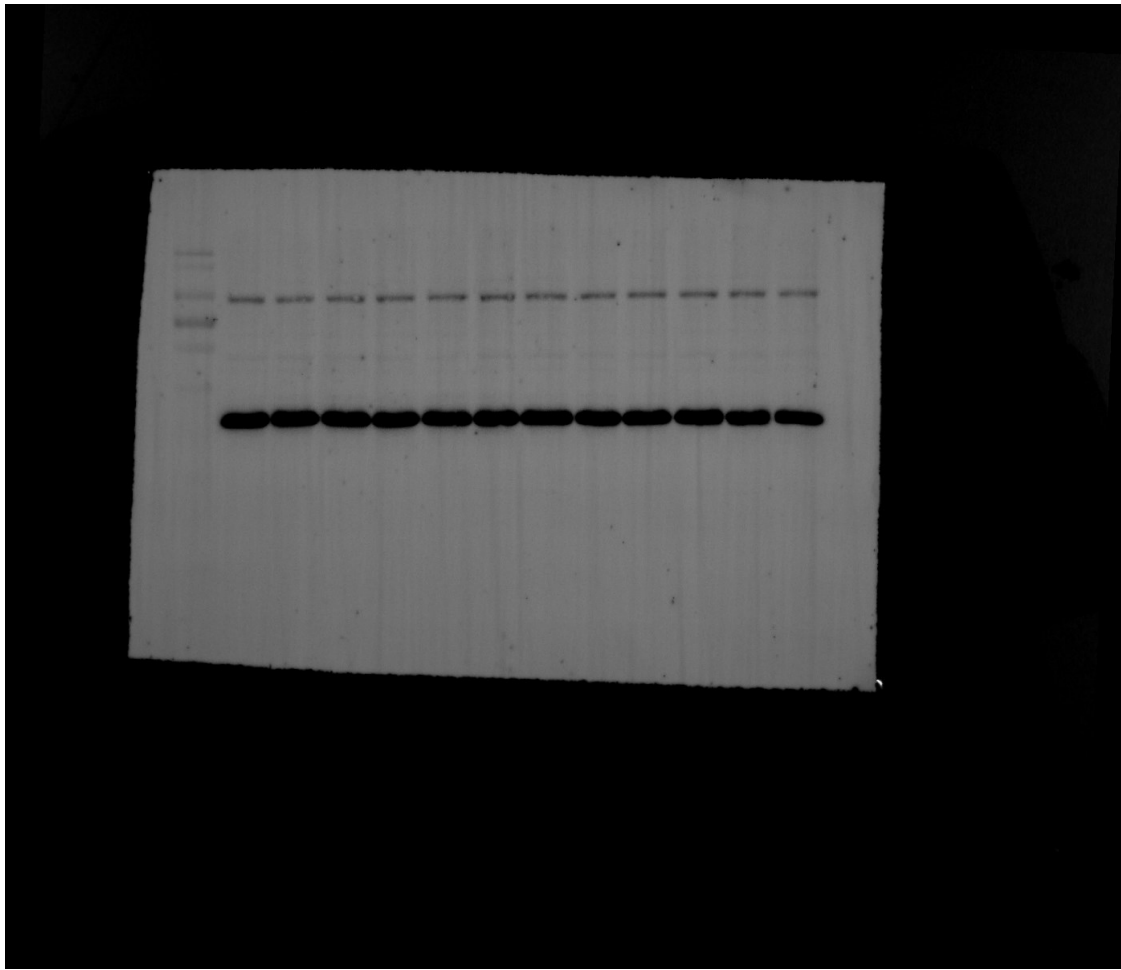

**Figure 5A**

p-p65

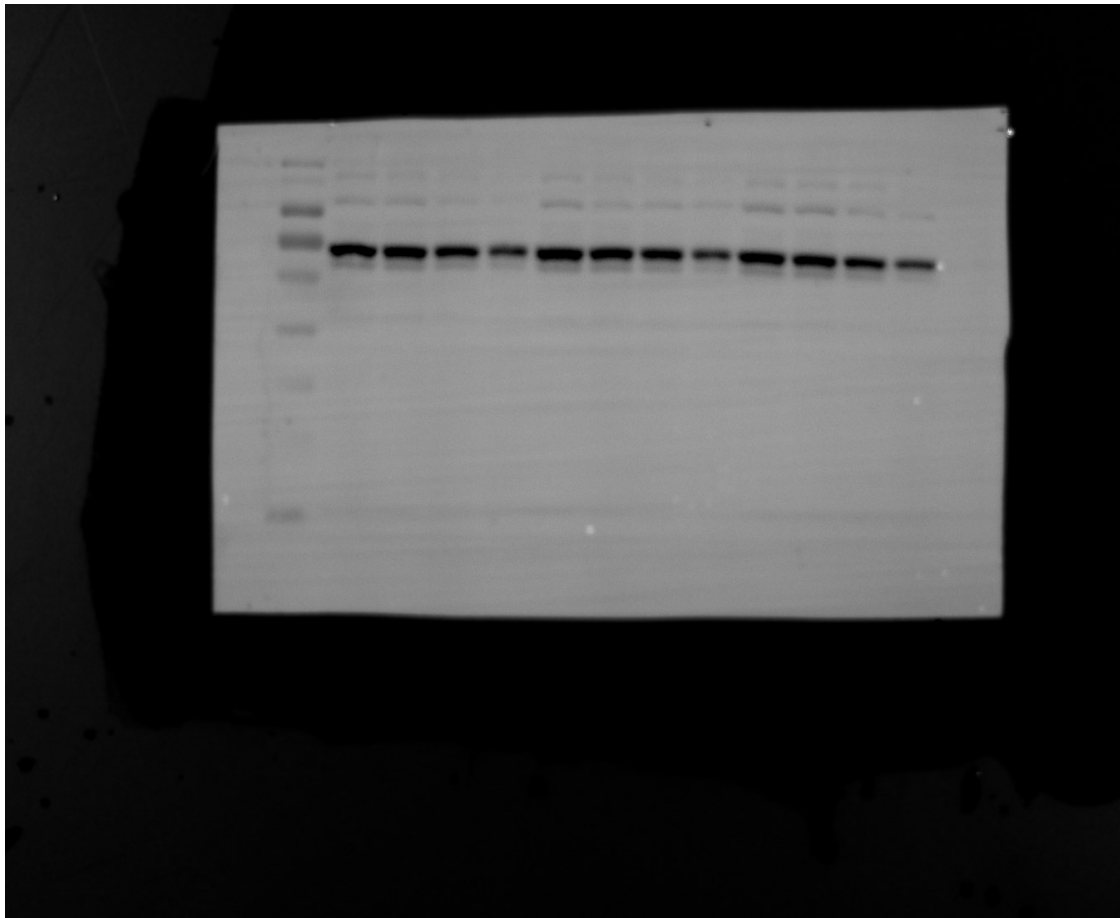

p65



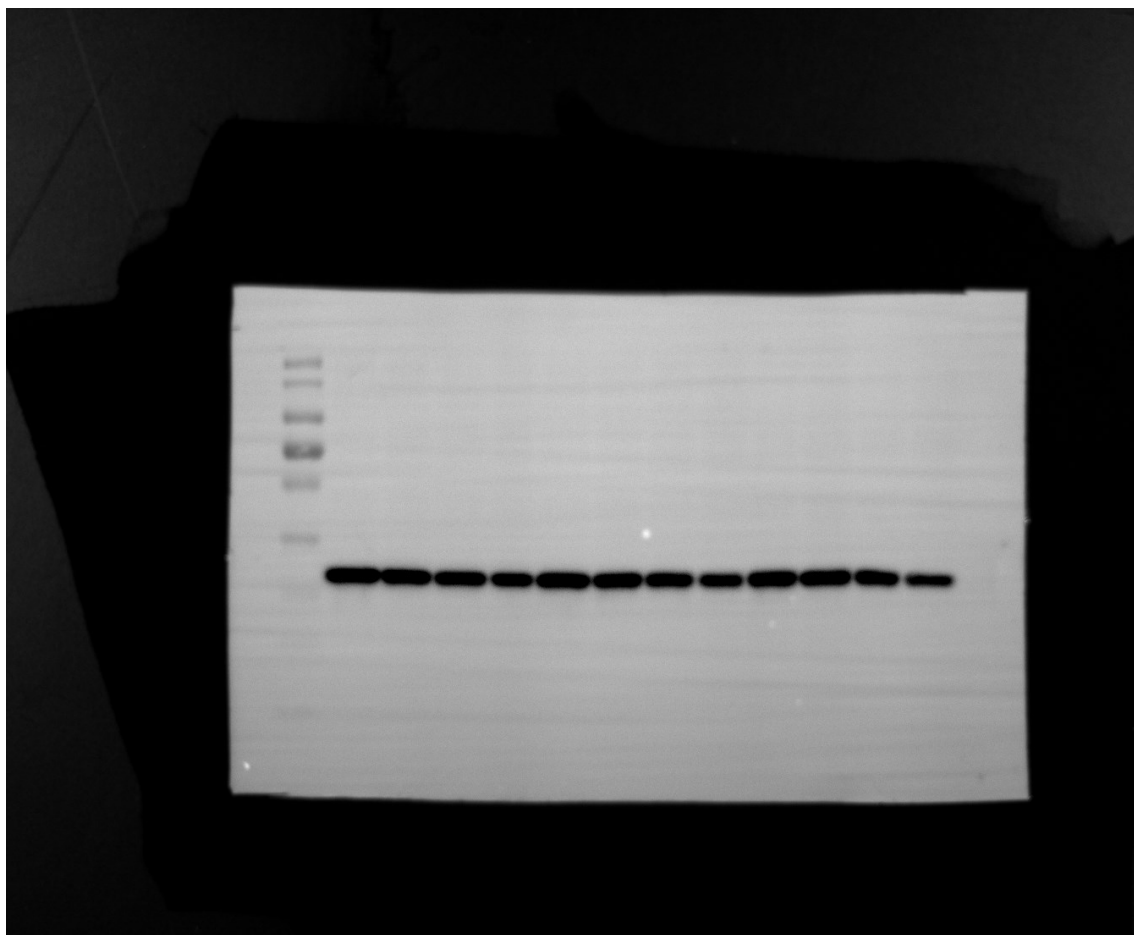

I $\kappa$ B $\alpha$

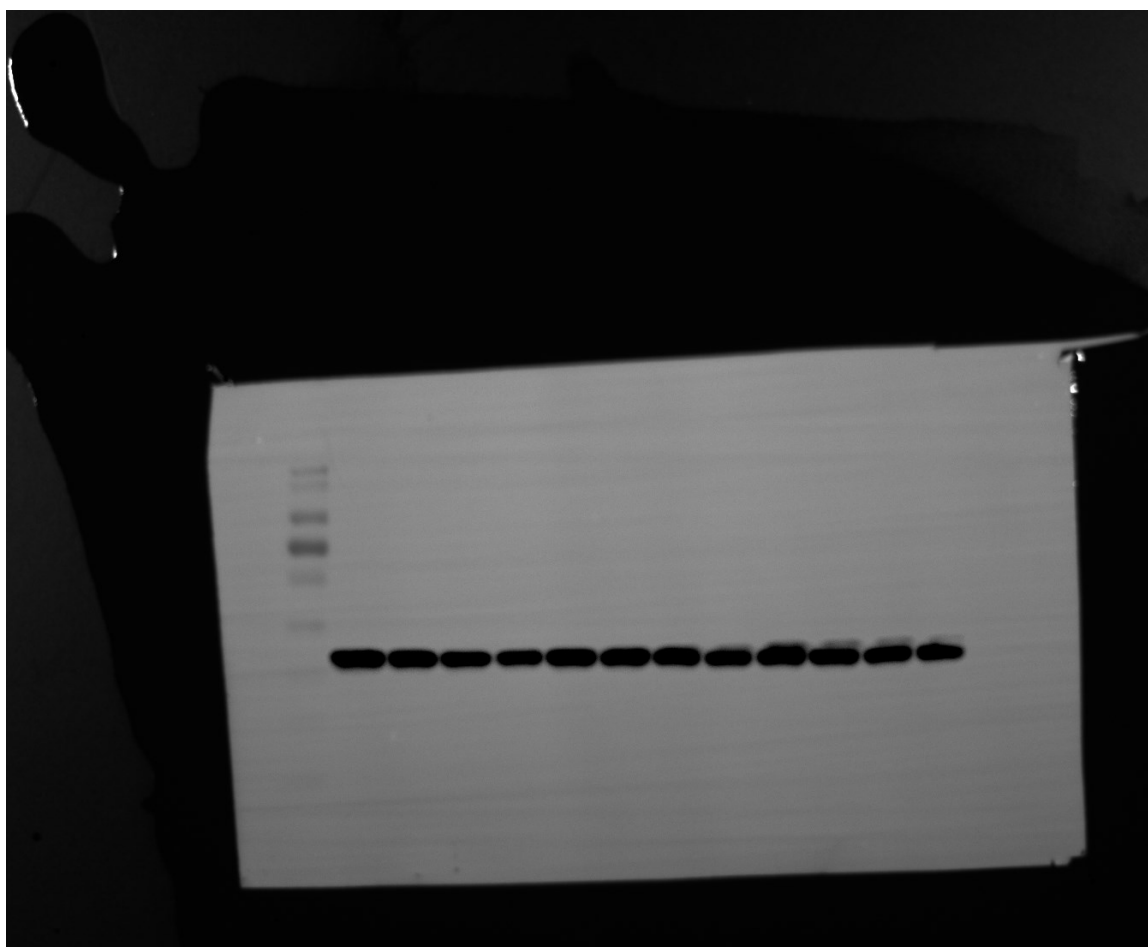

GAPDH

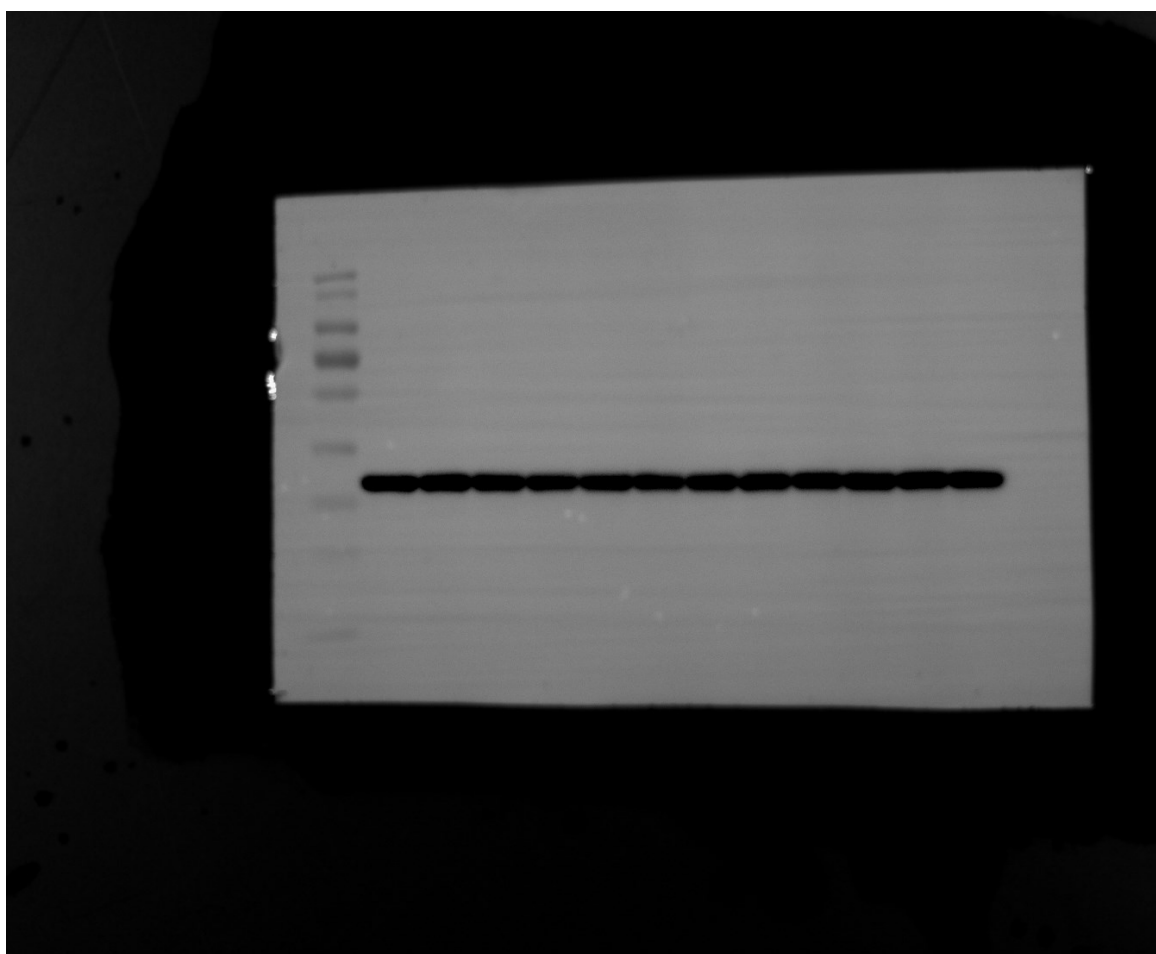

Supplement: Supplementary file 2 — Supplementary Material 2 [file 41598_2026_45671_MOESM2_ESM.pdf]
